# Delivery of Natural Agents by Means of Mesoporous Silica Nanospheres as a Promising Anticancer Strategy

**DOI:** 10.3390/pharmaceutics13020143

**Published:** 2021-01-22

**Authors:** Khaled AbouAitah, Witold Lojkowski

**Affiliations:** 1 Laboratory of Nanostructures and Nanomedicine, Institute of High Pressure Physics, Polish Academy of Sciences, Sokolowska 29/37, 01-142 Warsaw, Poland; 2 Medicinal and Aromatic Plants Research Department, Pharmaceutical and Drug Industries Research Division, National Research Centre (NRC), 33 El-Behouth St., Dokki 12622, Giza, Egypt

**Keywords:** mesoporous silica nanoparticles, controlled release, drug delivery systems, anticancer natural prodrugs, natural products, cancer targeting, nanoformulations/nanomedicine applications

## Abstract

Natural prodrugs derived from different natural origins (e.g., medicinal plants, microbes, animals) have a long history in traditional medicine. They exhibit a broad range of pharmacological activities, including anticancer effects in vitro and in vivo. They have potential as safe, cost-effective treatments with few side effects, but are lacking in solubility, bioavailability, specific targeting and have short half-lives. These are barriers to clinical application. Nanomedicine has the potential to offer solutions to circumvent these limitations and allow the use of natural pro-drugs in cancer therapy. Mesoporous silica nanoparticles (MSNs) of various morphology have attracted considerable attention in the search for targeted drug delivery systems. MSNs are characterized by chemical stability, easy synthesis and functionalization, large surface area, tunable pore sizes and volumes, good biocompatibility, controlled drug release under different conditions, and high drug-loading capacity, enabling multifunctional purposes. In vivo pre-clinical evaluations, a significant majority of results indicate the safety profile of MSNs if they are synthesized in an optimized way. Here, we present an overview of synthesis methods, possible surface functionalization, cellular uptake, biodistribution, toxicity, loading strategies, delivery designs with controlled release, and cancer targeting and discuss the future of anticancer nanotechnology-based natural prodrug delivery systems.

## 1. Introduction

In 2001, Vallet-Regi et al. [1] introduced a mesoporous silica material called MCM-41 that can be used as a drug carrier. The nanostructure (e.g., pore size) of MCM-41 can be optimized using different surfactants. Since then, many efforts and attempts have been made to synthesize versatile mesoporous silica nanoparticles (MSNs) with different nanostructures and morphologies to meet the demand for pharmaceutical and medical applications. The history of the synthesis of mesoporous silica materials dates back to 1992, when they were discovered by the Mobile Oil Corporation [2]. Silica is one of the most abundant minerals in the Earth’s crust and is also found in the food chain and the human body [3]. As a biomaterial, silica is extensively used in many applications such as dentistry, orthopedics, and dermatology. MSNs have a characteristic mesoporous nanostructure that offers many advantages for medical applications in disease diagnosis and therapy [4]. The unique features include easy synthesis, the possibility of various surface modifications, the ability to obtain a tunable particle size, uniform pore size, high surface area to pore volume, good biocompatibility, and chemical stability [5,6,7,8,9]. In addition, easy functionalization to achieve magnetic, fluorescent, and photothermal properties increases the chance of using MSNs in bioimaging. MSN nanostructures can provide excellent nanoplatforms to fabricate smart drug delivery systems (DDSs) with a high drug loading capacity and stimuli-responsive drug release effect compared to other nanocarriers [6,10]. Several nanocarriers have been used to deliver and control drug release, including niosomes, liposomes, dendrimers, lipid nanoparticles, and polymeric nanoparticles, but most of them have low stability and need external stabilization during synthesis. In contrast, MSNs have a strong Si-O bond that makes them stable (chemically and mechanically) to external responses in the surrounding environment [11,12,13]. It is generally accepted that encapsulation of drugs or therapeutic agents into MSNs can enhance their therapeutic activity, solubility, and bioavailability, as indicated by many studies [14,15,16,17,18,19,20].

A consequence of these advantages is that MSNs have gained much attention and popularity in DDSs during the last few decades for the delivery of cargo to specific sites in the organism. A large number of in vivo studies indicate the high biocompatibility/safety profile and low toxicity of MSNs if they are synthesized using an optimized way [21,22,23]. A careful optimization process is needed because many details of the nanostructure of engineered MSNs, i.e., size, shape, surface, presence of surfactant, and other factors like dose, administration route affect the safety profile. According to many animal studies, the toxicity of MSNs can be diminished by optimizing the synthesis parameters and surface modification, resulting in safe nanoparticles [24,25]. 

The administration route is an important characteristic for constructing any DDS. MSNs can be applied via different routes, including oral and intravenous injection [26,27,28,29,30]. Many choices in the development of pharmaceutical formulations depend on the target tissues and organs in the human body. An important advantage of DDS-based MSNs is that the amorphous forms of silica and silicates are generally recognized as safe materials for use as oral delivery ingredients (up to 1500 mg per day) according to the US Food and Drug Administration and the European Food Safety Authority [27]. MSNs are promising materials because they exhibit low toxicity levels in animals when applied, i.e., orally, injection [31]. 

The global market for nanomedicine accounts for 5% when novel nanomedicines translated from the lab to the clinics are concerned [32]. Recently, the first clinical trial in humans was conducted with oral delivery of fenofibrate formulation based on the ordered mesoporous silica [33]. 

Despite these promising results for nanotechnology application in building DDSs, most research for targeted cancer therapy has been focused on drugs and therapeutic molecules of a synthetic nature. Combating cancers with synthetic drugs is an established therapy, however, progress in this area of medicine is slow and the treatments are frequently associated with undesirable effects: side effects and also insufficient patient compliance. For this reason, extensive research is carried out to apply natural prodrugs (known also as natural products and natural agents) in anticancer therapies. 

Nature is a huge source of therapeutic substances, which can be derived from plants, microbes, and animals. Natural medicines account for 60% of anticancer agents used in clinical applications [34]. For example, vincristine, taxanes, and camptothecin are used in the treatment and prevention of cancer. There are still hundreds of promising new active natural anticancer agents to be discovered and renewed for cancer therapy [35,36,37]. The main advantages to using and developing natural prodrugs are that they offer safe, cost-effective, and have versatile pharmacological properties [38]. The main limitations for their use in cancer therapy are their poor water solubility, low bioavailability, short half-life, and non-specific targeting. 

Nanotechnology offers many ways to overcome these obstacles [39,40,41,42,43,44]. Natural pro-drugs can be embedded into MSNs, which can serve as effective nanocarriers for the delivery of anticancer natural prodrugs to target cancers. In this review, we present an overview of synthesis methods, surface functionalization, as well as biodistribution, biocompatibility, toxicity, biological performance. Additionally, drug loading and release strategies, and active targeting approaches for MSNs will be addressed. We also discuss delivery and controlled release systems for selected prodrugs using MSNs.

Available data provide considerable evidence that MSNs allow the limitations associated with prodrugs, such as poor water solubility, poor bioavailability, and low specific targeting ability, to be overcome. Compared to organic delivery systems (e.g., lipid nanoparticles, polymeric nanoparticles) [45,46], the delivery of natural prodrugs by means of MSNs allows high drug loading and permits multifunctional delivery or co-delivery systems. Generally, MSNs allow long-term release compared to organic nanoparticles. This is because the prodrugs are trapped inside nano-pores. In the case of encapsulation of prodrugs into organic nanoparticles, fast degradation of the organic substance leads to quick pro-drug release. The MSN-based nanomedicine technology is mature enough to be extended to thousands of prodrugs not yet investigated in clinical applications. 

To the best of our knowledge, this is the first review considering MSNs as delivery systems for anticancer natural prodrugs. The need for such a review is a consequence of rapid development in the field. This review may help researchers accelerate research and development of this important field of nanomedicine and, ultimately, clinical applications. 

## 2. Synthesis of Mesoporous Silica Nanostructures

**Figure 1:**
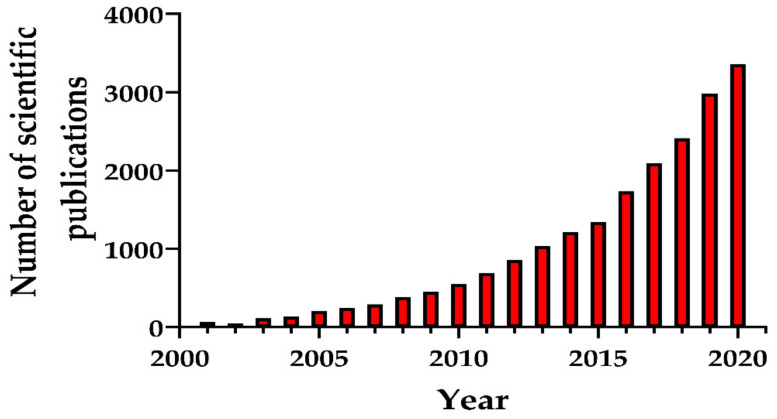
Number of scientific publications (research papers, reviews, book chapters) during the period 2001–2020 found by entering key words “mesoporous silica nanoparticles and synthesis”. The search was performed in ScienceDirect 10 September 2020.

Numerous synthesis methods have been developed to obtain MSNs with different morphological, structural, and pore geometry. Particular attention was paid to the production of biocompatible MSNs for medicine. Figure 1 presents the number of scientific publications (research articles, review articles, and book chapters) as an indicator of the growth in MSN synthesis methods due to their emergence as nanostructures for various promising applications.

### 2.1. Discovery, Synthesis, and Properties of MSNs

Porous materials (natural or artificial) are characterized by the presence of pores, including cavities, channels, or interstices. The properties of these materials vary depending on the characteristics of their pores: size, arrangement/structure, shape, porosity, and chemical composition. They have been extensively studied in different areas, including water purification, gas separation, catalysts, energy storage, adsorbents, electronics, engineering, tissue engineering, and drug delivery systems, among others [47]. Depending on the predominant pore size, the International Union of Pure and Applied Chemistry (IUPAC) classifies porous materials into three categories as shown in Table 1 [48,49].

**Figure 2:**
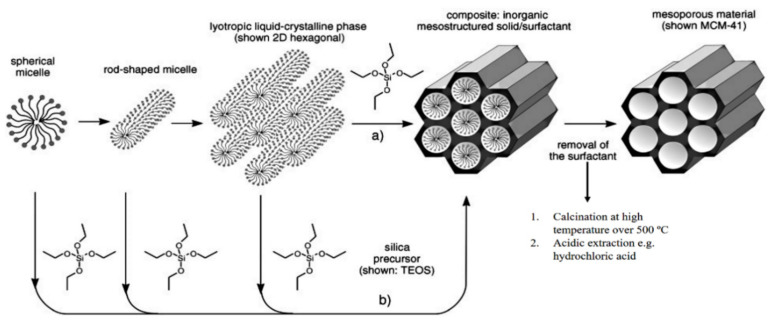
The formation mechanism for mesoporous materials by structure-directing agents. (**a**) True liquid–crystal template mechanism. (**b**) Cooperative liquid–crystal template mechanism. Reproduced with permission from [52], WILEY-VCH Verlag GmbH and Co. KGaA, 2006.

**Figure 3:**
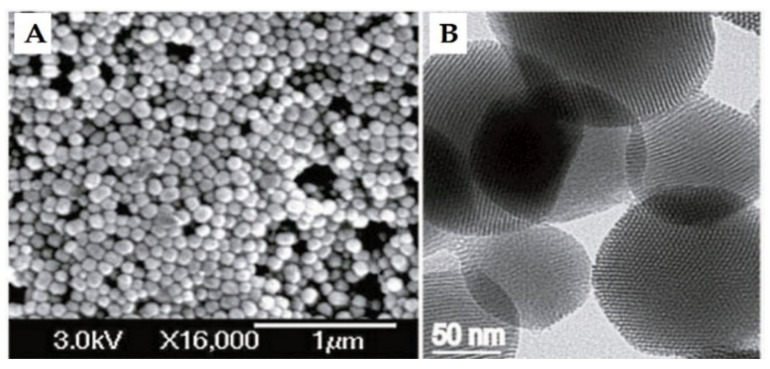
(**A**) Scanning electron microscopy (SEM) and (**B**) transmission electron microscopy (TEM) of MCM-41 material. Reproduced with permission from [23], Wiley-VCH Verlag GmbH and Co. KGaA, 2010.

The history of MSN materials dates back to the early 1990s, when the Kuroda group at Waseda University and researchers from the Mobil Company discovered Mobil crystalline materials (MCMs), nanoparticles with a hexagonal porous structure [2]. In 1992 with the discovery of MCM-41, a material prepared using the cooperative assembly of surfactant with silicates, a breakthrough in the area of ordered mesoporous structures and their successful preparation occurred [50,51]. In addition, an ionic template, such as cetyltrimethylammonium bromide (CTAB), could be employed as a structure-directing agent to produce MCM-41 and MCM-48 with pore sizes of 2 to 10 nm [50,51]. MCM-41 has a hexagonal pore shape and MCM-84 has a cubic pore shape. For DDSs purpose, MCM-41 is considered to be one of the most widely explored materials. The synthesis mechanism for MCM-41 is shown in Figure 2 and electron microscope images in Figure 3.

In 1996, another kind of MSN was discovered that has a non-ordered pore structure, named KIT-1 (Korea Advanced Institute of Science and Technology Number 1) [53]. The KIT family currently has many members, such as KIT-6, which has a hexagonal arrangement of pores [54], and KIT-5, which has a cubic ordered structure [55]. In 1998, the SBA-15 type (pore size 4–6 nm) MSNs introduced by Santa Barbara Amorphous (SBA), which have a hexagonal or cubic pore structure, were developed by means of nonionic surfactants in acidic conditions [56]. The cubic SBA-11, 3D hexagonal SBA-12, hexagonal SBA-15, and SBA-16 are mainly prepared based on non-ionic triblock copolymers, such as alkyl poly(ethylene oxide) (PEO) oligomeric surfactants and poly(alkylene oxide) block copolymers [10]. The typical synthesis of SBA-15 is dependent on tetramethyl-orthosilicate (TMOS) or tetraethyl-orthosilicate (TEOS) as the silica precursor reacting with a series of block-copolymer surfactants as structure-directing agents. The MCM and SBA materials are recognized as the first generation of hexagonally ordered pore structures and are the common MSNs used in research. A variety of strategies have been designed to attain tunable pore sizes (from less than 2 nm up to 30 nm). In this scenario, the adjustments are made depending on the surfactant template’s properties [57], pore swelling agents, such as mesitylene [50], or hydrothermal treatments [58]. 

**Figure 4:**
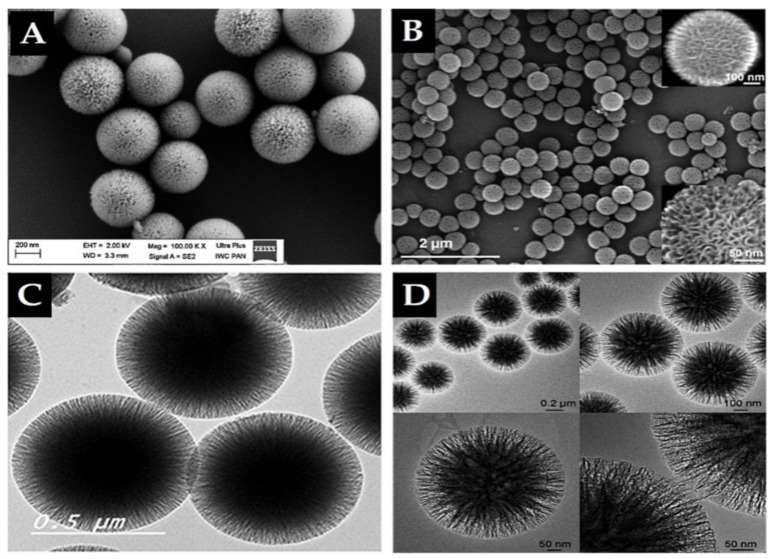
Electron microscope images of prepared KCC-1 material. (**A**,**B**) Scanning electron microscopy (SEM). (**C**,**D**) Transmission electron microscopy (TEM). Note, the dendritic fibrous 3D mesopore structure is clearly seen by SEM in B. A and C reproduced from [42,43], Impact Journals, 2018 and MDPI, 2020. B and D reproduced with permission from [59], WILEY-VCH Verlag GmbH and Co. KGaA, 2010.

Importantly, in 2010, high surface-area silica nanospheres with a fibrous morphology and non-ordered pore structure were discovered by a research group of the Catalysis Center at King Abdullah University of Science and Technology (KAUST Catalysis Center, KCC) [59]. This material, KCC-1, features a high surface area due to the presence of dendrimeric silica fibers and their respective channels, making KCC-1 a first-of-its-kind material. It is a spherical particle with 3D tomography, a uniform size ranging from 250 nm to 500 nm, high surface area, and large pore size in a non-ordered structure (Figure 4). Synthesis of KCC-1 [59] was accomplished by a microwave-assisted, templated, solvothermal strategy using cetylpyridinium bromide (CPB) or cetyltrimethylammonium bromide (CTAB) as a surfactant (template), 1-pentanol as a co-surfactant, TEOS as the silica source, urea (catalyst-hydrolyzing agent), and a mixture of the cyclohexane solvent and water (as the reaction solvent). The chemicals were introduced to the reaction system stepwise with mixing and microwave-assisted heating applied (in a closed vessel >1200 °C) for a predetermined time for the reaction. Finally, the solution was filtered or centrifuged, washed, and the obtained material calcinated at high temperature (>550 °C). Many research groups changed the surface of substances used in the synthesis in addition to the parameters. For example, Bayal et al. [60] showed that changing the concentrations of urea, surfactant (CTAB instead of CPB), or solvent (1-pentanol), the reaction time, or temperature can result in various particle sizes, fiber densities, surface areas, and pore volumes for KCC-1. Such easy manipulation and controlled synthesis of this material make KCC-1 a good solution for versatile applications in the environment, energy, biology, medicine, and other fields [42,43,61,62,63,64,65,66,67,68]. KCC-1 could be recommended for different small or large drug/therapeutic agents, possibly for any design and pathological disorder due to KCC-1 s unique physicochemical features. Our research team is among the first to study KCC-1 for DDSs [42,43,68,69], and we think that research on KCC-1 will increase soon. In the literature, there are references to “spherical wrinkled mesoporous silica” (WMS) [70,71,72] and KCC-1 is known also “dendritic fibrous nano-silica” (DFNS) [73]. They were all obtained based on changing the synthesis conditions and parameters of the original synthesis method for KCC-1 particles.

Unlimited opportunities exist for the synthesis of MSNs in pure, doped, composite, and modified forms by employing different templates (soft and hard), conditions, and methods [74]. 

Due to the unique properties of the KCC-1 family, they offer a wide range of possible applications. It seems that KCC-1 has comparable potential as the commonly used members of the MCM and SBA families, as well as Stober silica, solid silica discovered before all the families [73]. Table 2 presents the major physicochemical properties for fibrous KCC-1, MCM-41, SBA-15, and others. Below, we highlight the common and promising families that could be favored for drug delivery and medical applications. Numerous interesting review articles have been published on MSN synthesis strategies and applications that we recommend for further reading [10,22,32,73,75,76,77,78,79,80,81,82,83].

### 2.2. Surface Modification of MSNs for Drug Delivery

The keystone in the development of DDSs is to functionalize their surface [84,85] to increase their drug loading and release, leading to high therapeutic effects. The surface chemistry modulates the interaction of MSNs with the surrounding media. The MSNs have a high density of silanol groups (Si-OH) on their surface, allowing surface modification by various organic functionalities (e.g., silanes, polymers, proteins, and targeting moieties). Thus, MSNs can load various drugs with high capacity and release them in a sustained or controlled manner. A variety of functional groups can be used, such as amine, carboxylate, phosphonate, polyethylene glycol, octadecyl, thiol, carboxylic acid, and octadecyl groups. To introduce functional groups on the surface of MSNs, covalent bonding and electrostatic interactions are generally used [86]. The common approach to modify MSNs is to use organic silane groups via direct covalent attachment by means of co-condensation or post-synthetic grafting. 

**Figure 5:**
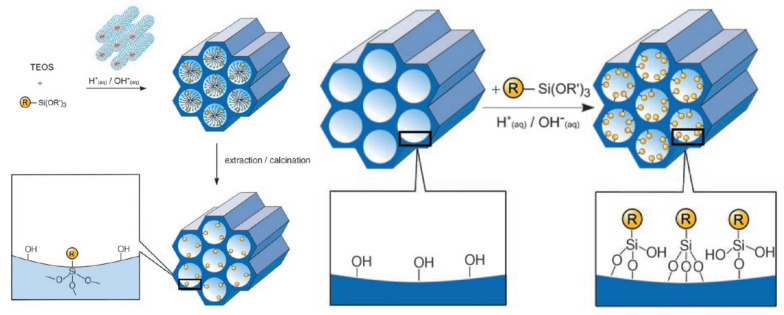
A schematic presentation of the organic functionalization methods for mesoporous silica materials. (**A**) Co-condensation method and (**B**) grafting method. Reproduced with permission from [52], WILEY-VCH Verlag GmbH and Co. KGaA, 2006.

The co-condensation method is referred to as a one-pot synthesis method [87,88] as presented in Figure 5A. The desired functional group of silanes, such as 3-aminopropyl-triethoxysilane (APTES “NH_2_”) is added during the sol-gel synthesis process together with the silica source (e.g., TEOS). Next, the template is removed (Figure 5A) [52,87,89]. To remove the surfactant template, an extractive method using alcoholic/acidic solution under reflux can be used [90]. Removing the template anchors the organic residue covalently to the porous walls of the MSNs. This approach has the advantages of easy preparation, more homogeneous distribution of organic units, and high drug loading [52,83]. Despite these advantages, disadvantages are a potential change in the mesoscopic order, disordering the porosity and reducing the pore diameter, pore volume, and specific surface areas [52]. 

A post-synthetic approach refers to the subsequent modification of the inner/outer surface of MSNs by covalent and electrostatic interactions. The modification is usually achieved after surfactant removal from MSNs (Figure 5B). The most remarkable advantages of this approach are selective functionalization (either external or internal surfaces) and retention of the mesostructure of MSNs during synthesis. The major disadvantages include reduced pore size and non-homogeneous distribution of functional groups into/onto pores [52,91,92].

### 2.3. The Biological Performance of MSNs

#### 2.3.1. Cellular Uptake

Any nanocarriers have to cross the cell membrane boundary to enter cells, allowing the therapeutic effects of the delivered drugs. The internalization of nanoparticles carrying therapeutic agents into cells represents the initial step in successful drug delivery [93,94]. The acting mechanisms and surface chemistry of nanocarriers are the major parameters in designing a preferred DDS for any pathological disease [78]. Nanoparticles mainly access the cell interior via simple diffusion or translocation as an energy-dependent process [95]. The most common mechanism of their internalization is the energy-dependent endocytosis, which allows the uptake of nanoparticles and submicron particles from an extracellular environment to the cell plasma membrane [96]. The mechanisms can generally be classified into phagocytosis, pinocytosis, micropinocytosis, receptor-mediated endocytosis, clathrin-mediated endocytosis, caveolin-mediated endocytosis, and others (e.g., Arf-6, Rho-A or IL2Rb-dependent pathway, flotillin, or CDC42 (CLIC/GEEC)-dependent endocytosis) [93]. The intracellular uptake and trafficking mechanisms by which nanoparticles are internalized in cells vary broadly depending on many factors, including size, shape, charge, and surface modification. Therefore, these factors should be taken into consideration in constructing DDSs.

##### Size of MSNs

**Figure 6:**
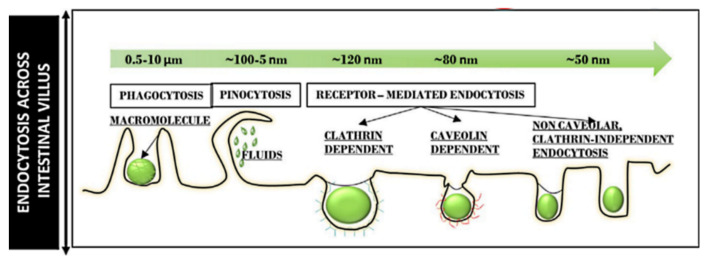
Different endocytosis pathways across the intestinal villus for particles of different sizes. Reproduced with permission from [97], Elsevier Inc., 2020.

Particle size determines the intracellular uptake of MSNs (Figure 6) [97]. It is generally accepted that particles with the smaller size of 50 nm can internalize into cells via non-phagocytosis [98]. Nanoparticles up to 150 and 200 nm in size are internalized by pinocytosis, such as clathrin-mediated endocytosis and caveolin-mediated endocytosis, respectively [99,100]. In contrast, particles from 250 nm to 3 μm in size can internalize the cells by macropinocytosis and phagocytosis [101]. It is also accepted that the microparticles are efficiently taken up through phagocytosis but the process depends also on other parameters, such as geometry, surface charges, and functional groups of microparticles [102]. Particles with sizes ranging from 30 to 50 nm internalize also efficiently via receptor-mediated endocytosis [103]. Despite extensive investigations exploring the relationship between particle size and uptake pathways, the results are inconsistent [101,104,105,106]. The main reason for such contradictions can be attributed to the complexity of control of structural parameters, such as shape and surface charges. For successful internalization, particles should avoid degradation (within endosomal/lysosomal vesicles) and release their cargo in the cytoplasm [107]. Therefore, particle size is important in tailoring DDSs. It is also important for their intersections with the reticulo-endothelial system (RES), which is responsible for elimination of nanoparticles from the body, and prolong the circulation time in the blood. In this context, several studies have shown that increasing the particle size increases clearance from the body, reducing the therapeutic impact [108,109,110,111,112].

**Figure 7:**
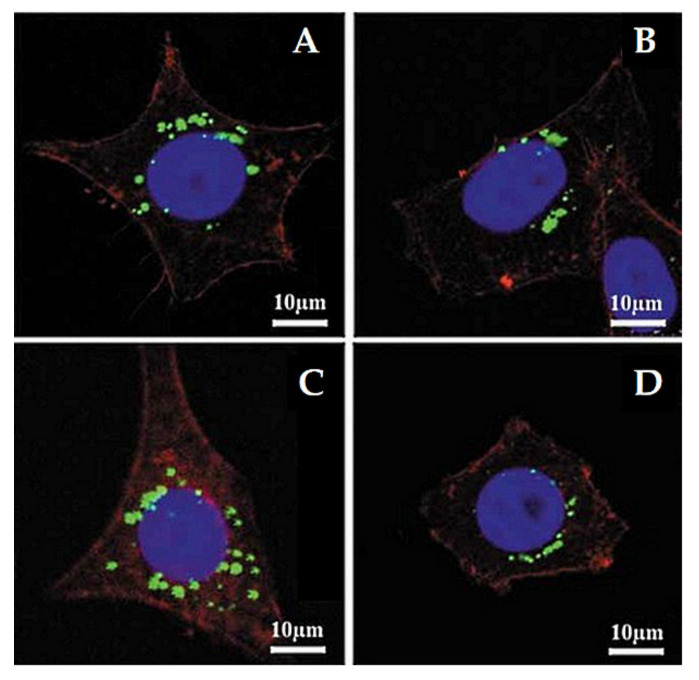
Confocal laser microscopy images of HeLa cells after incubation with different sizes of MSNs labeled with fluorescein isothiocyanate (FITC) green fluorescence (MSN-FITC) (100 µg mL^−1^, green) for 5 h at 37 °C. (**A**) 170 nm, (**B**) 110 nm, (**C**) 50 nm, and (**D**) 30 nm. The cell skeleton was stained with rhodamine-phalloidin (red), and the cell nucleus with 4′,6-diamidino-2-phenylindole (DAPI; blue). Reproduced with permission from [103], WILEY-VCH Verlag GmbH and Co. KGaA, 2009.

**Figure 8:**
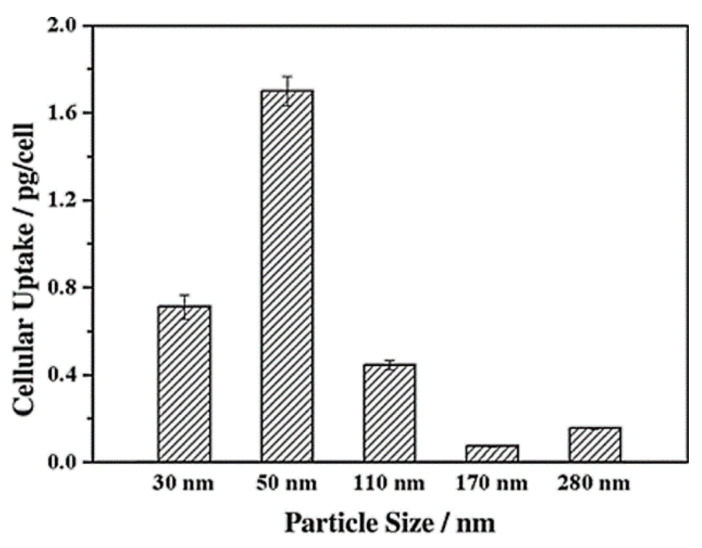
Cellular uptake of FITC-MSN-x based on nanoparticle size. Reproduced with permission from [103], WILEY-VCH Verlag GmbH and Co. KGaA, 2009.

Lu et al. [103] investigated the impact of various sizes (30, 50, 110, 170 nm) of MSNs on cellular uptake by HeLa cancer cells using MSNs labeled with fluorescein isothiocyanate (FITC) green fluorescence (MSN-FITC) and confocal laser scanning microscopy. They found that the MSNs were internalized as non-uniform green-fluorescent aggregates in the perinuclear region, and no MSNs penetrated the nucleus (Figure 7). Quantifying the internalization of MSNs, they concluded that the cellular uptake is highly particle size-dependent, observing the order 50 > 30 > 110 > 280 > 170 nm (Figure 8). Haddick et al. [113] demonstrated that MSNs with a size of 160 nm had the fastest cellular internalization in T24 bladder cancer cells through receptor-mediated cellular internalization compared to 60, 80, 100, and 130 nm, leading to the highest level of gene knock-down for antitumoral effects. Yang et al. [114] tested different sizes of rod-shaped SBA-15 (from 80 to 200 nm) and spherical MCM-41 particles, as well as their intracellular uptake in human osteosarcoma cancer cells (KHOS). They found that the cellular uptake efficiency depends on the particle size and shape.

##### Surface Charges of MSNs

Another critical factor influencing the cellular uptake of nanoparticles is the surface charge. MSNs are characterized by silanol groups permitting to add different functional groups, modifying their surface to be either cationic or anionic [115]. Most cells have a negatively charged cell membrane, enhancing the uptake of positively charged nanoparticles. Several studies have shown that positively charged nanoparticles internalize with higher uptake than neutral and negatively charged nanoparticles [116,117,118,119]. Furthermore, neutral nanoparticles usually have lower cellular uptake compared to negatively charged nanoparticles [98,120]. As a result of the internalization of nanoparticles by cells, their interaction with the cell membrane can occur by means of gelation of membranes (with negatively charged nanoparticles) or fluidity of membranes (with positively charged nanoparticles) [121,122]. On the one hand, the positively charged nanoparticles mainly enter cells via micropinocytosis; on the other hand, the negatively charged nanoparticles always enter cells by clathrin- or caveolae-independent endocytosis [123].

Positively charged MSNs generally exhibit higher endocytosis efficiency compared to negatively charged MSNs due to the higher affinity for the negatively charged cell membranes. Jambhrunkar et al. [124] prepared MCM-41 with negative and positive charges for delivering curcumin. They found that the positively charged MCM-41-NH_2_ had more efficient uptake in the human squamous cell carcinoma cell line (SCC25) than negatively charged particles. Baghirov et al. [125] studied spherical and rod-shaped MSNs that were either non-modified or modified with a poly(ethylene glycol)-poly(ethylene imine) (PEG-PEI) block copolymer in in vitro models of the blood–brain barrier. The results showed that the modified MSN-PEG-PEI particles exhibited robust uptake in RBE4 rat brain endothelial cells and Madin–Darby canine kidney epithelial cells. Our group performed a comprehensive study of cellular uptake using two types of MSNs: KCC-1 and MCM-41 (non-modified, positive charges with -NH_2_, and folic acid ligands) [42]. The FA-conjugated MSNs exhibited higher cellular uptake than MSNs-NH_2_ and non-modified MSNs.

##### Morphological Structures of MSNs

The morphological structures (i.e., different shapes) play an important role in the cellular uptake and trafficking of nanoparticles into cells or organs. Trewyn et al. [126] studied the impact of different MSN shapes on cellular uptake in vitro, finding that a tubular structure achieves more efficient uptake by both cancer and normal cells than those of spherical morphology. Huang et al. [127] investigated the effect of three differently shaped particles on non-specific cellular uptake by human melanoma (A375) cells. Their results proved that particles with a larger aspect ratio are efficiently internalized by cells in large amounts at faster rates. Another study tested the core–shell MSNs with spherical or rod-like shapes for cellular uptake, showing that a rod shape results in more internalization by cells than a spherical shape [128] It is generally accepted that this effect could be due to the larger contact area of the rod than a sphere, permitting high favored internalization of nanoparticles in cell membranes [116,128] Furthermore, rod-shaped MSNs exhibit superior intracellular uptake compared to spherical MSNs [129]. The shape of the nanoparticles can allow a specific mechanism of intracellular uptake. In this context, Hao et al. [130] reported that the spherical particles are taken up by cells via clathrin-mediated endocytosis, whereas the rod-shaped particles enter cells through caveolae-mediated endocytosis. 

##### Other Features of MSNs

One significant characteristic of any nanocarrier delivery system is hydrophobicity. Nanoparticles that have a hydrophobic nature exhibit a higher affinity for interacting with the cell membrane than those with a hydrophilic nature, contributing to improved cellular uptake [94].

#### 2.3.2. Biocompatibility and Biodistribution of MSNs

**Figure 9:**
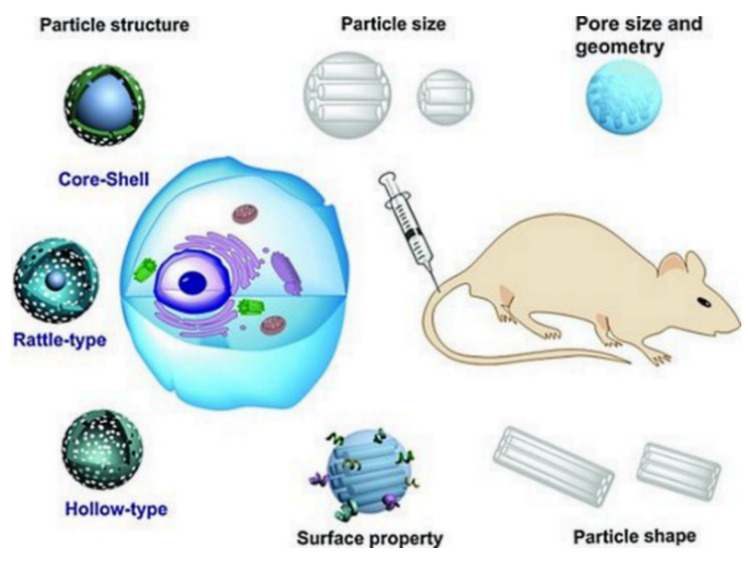
Schematic illustration of the biocompatibility and biotranslocation of MSNs and the main physical–chemical characteristics. These highly influence the cellular uptake, intracellular translocation, and cytotoxicity on the in vitro level, and the biodistribution, biodegradation, excretion, and toxicity on the in vivo level. Reproduced with permission from [131], WILEY-VCH Verlag GmbH and Co. KGaA, 2012.

Any DDSs introduced into clinical investigations should exhibit biocompatibility with body tissues and organs. The biocompatibility is dependent on many MSN characteristics, such as size, shape, surface functionality, porosity, route of administration, and structure (Figure 9) [131].

Most animal studies indicate the high biocompatibility and safety of MSNs [31,132,133]. The degree of biocompatibility of MSNs can vary according to many factors such as synthesis conditions, suitable structural features, and appropriate route at the right dosage [8,133,134,135,136,137]. As with other nanomaterials, for future translation to clinical applications, the safety aspects of MSNs should be considered carefully for each type [133]. Below, we present some studies highlighting the biocompatibility of MSNs in vitro and in vivo. Park et al. [138] investigated the biodistribution and biocompatibility of MSNs intravenously injected into mice at 20 mg/kg. The histopathological examination showed no significant toxicity compared to the control group. Their studies also indicated that MSNs are mostly cleared from the liver, spleen, heart, kidneys, brain, and lungs after 4 weeks. Hudson et al. [139] examined the biocompatibility of non-modified MSNs with particle sizes of ~150 (pores about 3 nm), 800 nm (pores about 7 nm), and ~4 µm (pores about 16 nm) at different does/concentrations. In vitro results in mesothelial cells showed that the cytotoxicity depends on the concentration; increasing concentration increases cytotoxicity towards cells. For in vivo studies, mcice were injected (intra-peritoneal, intra-peritoneal, and subcutaneous) at single dose of 30 mg/mL per mouse. The biocompatibility of MSNs in vivo depends on the dose and the route of administration. The subcutaneous injection of MSNs in rats indicates good biocompatibility, whereas intraperitoneal and intravenous injections at very high dose ~1.2 g/kg is lethal for mice due to toxicity or distress necessitating euthanasia, but at dose of ~40 mg/kg is safe. This severe systemic toxicity can be mitigated by further surface modification of the MSNs. Lu et al. [23] evaluated various doses of MSNs intravenously injected in mice (twice per week) for 14 days, they concluded that dose at 50 mg/kg is well tolerated in mice, no toxicity, no apparent abnormalities on the histopathological level or lesions were observed. They also revealed that this dose is adequate for the pharmacological application in cancer therapy.

**Figure 10:**
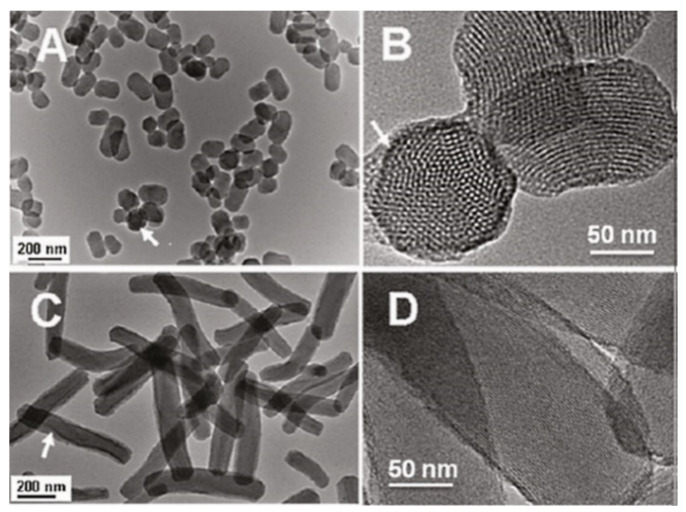
Characterization of short rod MSN labeled with FITC (NSRFITC) and long rod MSN labeled FITC (NLRFITC). (**A**) TEM image of NSRFITC. (**B**) TEM image showing the mesostructure of NSRFITC. (**C**) TEM image of NLRFITC. (**D**) TEM image showing the mesostructure of NLRFITC. Arrows denote FITC embedded in a particle. Reproduced with permission from [30], American Chemical Society, 2011.

**Figure 11:**
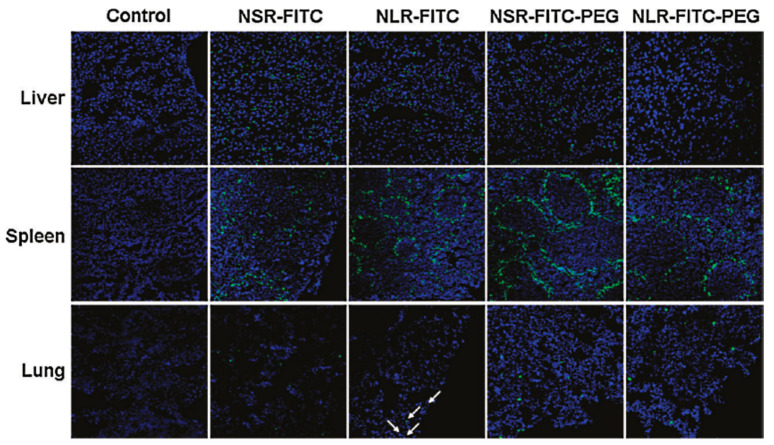
Biodistribution of differently shaped and poly(ethylene glycol) (PEG)ylated MSNFITC in liver, spleen, and lung observed by confocal microscopy 2 h after intravenous injection. Arrows denote NLRFITC distribution in the lung. Reproduced with permission from [30], American Chemical Society, 2011.

**Figure 12:**
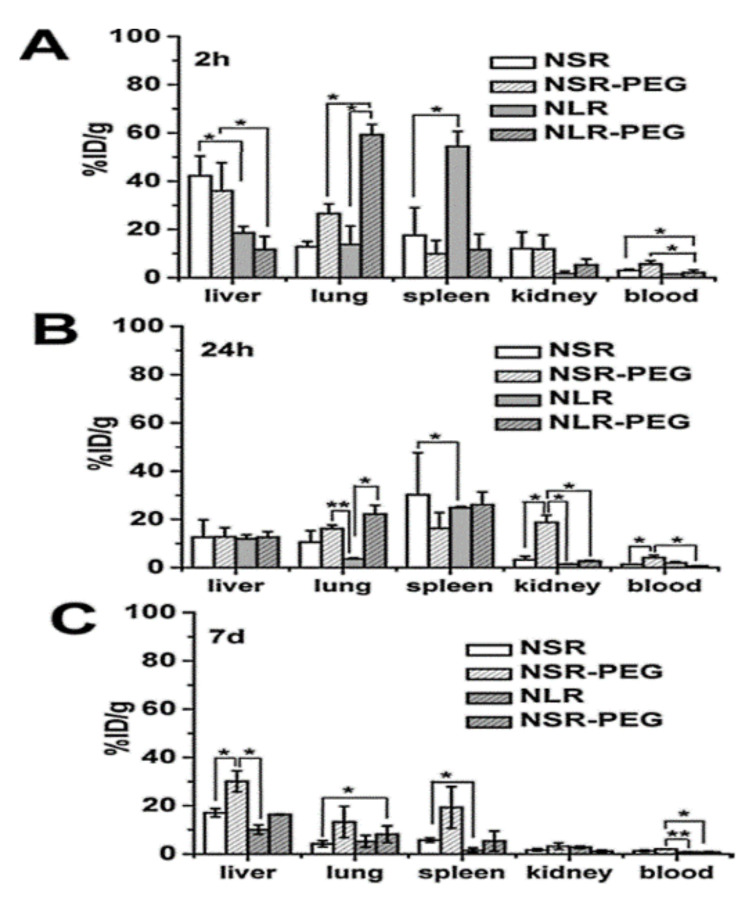
Quantitative analysis of differently shaped and PEGylated MSNs in organs and blood by ICPOES. Relative Si contents in liver, spleen, and kidney at (**A**) 2 h, (**B**) 24 h, and (**C**) 7 d post-injection. Data are the mean ± SD from three separate experiments. * *p* < 0.05; ** *p* < 0.01 for the comparison of Si contents of differently shaped and PEGylated MSNs in organs and blood. Reproduced with permission from [30], American Chemical Society, 2011.

Huang et al. [30] evaluated the biocompatibility of differently shaped and PEGylated MSNs (Figure 10, Figure 11 and Figure 12), measuring various blood and serum biochemical indicators 24 h and 18 days after injection of MSNs at a dose of 20 mg/kg. All hematology markers were within normal ranges without any considerable toxicity, showing excellent biocompatibility. The results indicated that these particles do not influence liver function, and other parameters were also in the normal range. Concerning the quantitative determination of biodistribution and clearance, approximately 80% of MSNs are trapped in RES of the liver, spleen, and lung after 2 h of administration. Comparing the Si contents of different organs (at 2 h, 24 h, and 7 days), the Si content obviously decreased over time, indicating the possible degradation and clearance of MSNs from the liver, spleen, lung, and kidney. Moreover, the circulation time of MSNs in blood shows that long rod MSN (NLR) has a longer blood circulation time than short rod MSN (NSR), and the effect of surface modification by PEGylation is partially dependent on the shape.

Yildirim et al. [140] evaluated the interactions of MSNs with different surface functional groups (ionic, polar, neutral, and hydrophobic) on blood parameters (hemolytic activity, thrombogenicity, and adsorption of blood proteins) to understand their biocompatibility. They concluded that the blood compatibility of MSNs positively improves with surface functional groups. Table 3 shows some data reported on the biocompatibility, biodistribution, and clearance of MSNs in vitro and in vivo.

#### 2.3.3. Toxicity of MSNs

**Figure 13:**
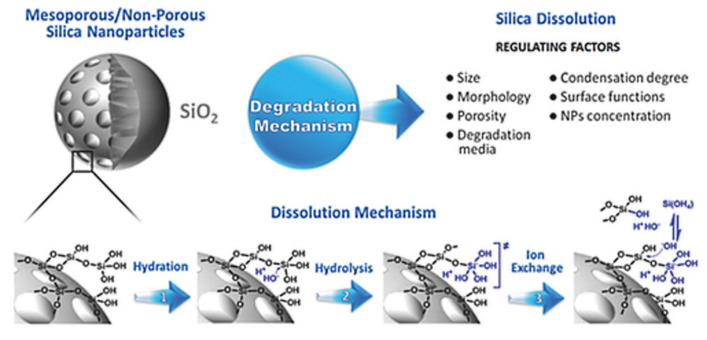
Schematic representation of the intact and degraded structures of silica material nanoparticles with the mechanisms and regulating factors underlying degradation. Reproduced from [155], WILEY-VCH Verlag GmbH and Co. KGaA, 2017.

For preclinical and further clinical investigations, nanocarriers should be optimized to avoid undesirable characteristics (e.g., toxicity, side effects, non-specific interactions) and to allow good biological performance [131]. As one of the most abundant materials on Earth, silica (or silicon dioxide) in crystalline form can be found in nature as sand or quartz [149]. In contrast, the amorphous form is present in biological materials, including plants, cells, microbes (e.g., bacteria), vertebrates, and invertebrates [150]. Silica is also endogenous to human tissues, such as cartilage and bone [151]. Several efforts are underway to identify the toxicity of both the crystalline and amorphous forms of silica in different methods of application [10]. Crystalline silica mainly results in toxicity as a result of breathing fine crystalline powders created by the extraction of stone materials in soil [86]. Because it is found in vegetables, whole grains, and seafood, silica is a considerable part of the human diet (approximately 20–50 mg silicon/day for Western populations and reaching 200 mg/day for people whose diet is mainly plant-based as in China and India) [152]. Furthermore, after ingestion of silica, it circulates in the blood plasma and is absorbed in the form of silicic acid; up to 41% of silicic acid is excreted in the urine [153]. Silica nanomaterials are hydrolytically unstable and dissolve into the soluble form of silicic acid (Si(OH)4, pKa 9.6) [152]. This can occur through three different processes: hydration, hydrolysis, and ion-exchange [154]. A schematic representation of silica degradation is shown in Figure 13 [155]. Silicic acid has good bioavailability, contributing many health benefits, such as maintaining bone health [154,156,157]. The FDA has approved silica as “generally recognized as safe” for use in food additives and pharmaceutical products [86,155]. Silica nanoparticles have also been approved by the FDA for cancer imaging in clinical trials [158] and MSNs being developed with high potential for DDSs in clinical investigations [159]. 

The biosafety of engineered MSNs has been confirmed by several studies. As shown in the literature, MSNs have insignificant toxicity, and the degree of toxicity is identified as low from in vivo studies. Additionally, even such insignificant toxicity can be reduced with the optimization of the synthesis process. However, a few reported data [160,161,162,163] provide contrary reports. The plausible reason for this is that there are many factors affecting the biocompatibility and safety of MSNs (e.g., shape, size, surface functional groups, physicochemical properties). For example, the method of removing the surfactant/template after MSNs synthesis (by calcination or by refluxing) influences the final cytotoxicity [139]. According to a number of in vivo experiments, a coherent message regarding the toxicity of MSNs is that that the toxicity depends on the dose/concentration used. For example, Hudson et al. [139] investigated the toxicity for MSNs (single dose) in vivo, they evaluated various doses and administration routes. They concluded that a very high dose (1.2 g/kg) is lethal for mice compared to the half dose which is well-tolerated and safe when applied by intraperitoneal or intravenous injection. Liu et al. [164] studied the single and repeated dose of MSNs via intravenous administration in mice. In the single-dose toxicity investigations, they found that the LD50 is higher than 1000 mg/kg. They also demonstrated that the groups that received low doses of MSNs did not show any behavioral, hematology, and pathological changes, whereas the groups that received high doses (1280 mg/kg) did not survive. In the repeated dose toxicity experiments, the mice groups were given continuously for 14 days followed by observation for a month. The results display that no mortality and no remarkable changes (in pathology or blood parameters) were detected. They also reported that the treatment of MSNs at daily doses (80 mg/kg) for 14 days is safe without any adverse effects in animals. Fu et al. [29] evaluated toxicity of MSNs (110 nm) in ICR mice treated by different routes: hypodermic, intramuscular, intravenous injections, and oral administration. They found that the oral route is well tolerated in mice even when increased to 5000 mg/kg compared to the intravenous route which shows the least threshold. As such results and others available from literature generated evidence to show that MSNs are well tolerated and safe in animals by various routes of administrations, i.e., oral, and intravenous injections [29,133,164,165]. However, there is no doubt that optimized production of MSNs and the final nanoformulation can achieve good biocompatibility and safe nanoparticles for treating diseases. Table 4 lists some studies that have explored the toxicity of MSNs and their delivery systems. For more reading concerning the toxicity and biosafety of MSNs, there are several extensive reviews [10,137,151,166,167,168]. The toxicity of any material/object, including MSNs, in a given environment is dependent on the dose [168]. As reviewed by Croissant et al. [168], there are mainly two mechanisms governing the toxicity of MSNs on the cellular level [88]. The first mechanism is surface silanolates that lead to membranolysis after the electrostatic interactions between MSNs and phospholipids of the cell membrane occur [169]. The second mechanism is reactive oxygen species (ROS) generation, which leads to cell death (by necrosis or apoptosis) by means of membranolysis [170]. Reducing the possible toxicity and improving the biosafety of MSNs can be achieved by optimizing the synthesis properties of MSNs for drug delivery and biomedical applications.

## 3. Drug Loading and Release Strategies

### 3.1. Drug Loading Strategies

A unique feature of MSNs (e.g., large pore volume, high surface, pores, stability) makes them one of the most common nanocarriers exploited for drug delivery with a high drug loading capacity for a variety of drugs. Generally, drugs or therapeutic molecules can be loaded into MSNs with or without pore-capping. In the first technique without pore-capping, hydrophobic or hydrophilic therapeutic agents directly load MSNs with covalent or noncovalent bonding or electrostatic interactions. Loading of drugs or therapeutic agents into the mesopore network of MSNs delivers them to target tissues while simultaneously saving them from undesirable factors found in the surrounding environment (e.g., enzymatic degradation in the body) [9]. To load a suitable amount of drug, MSNs are immersed in the desired stock solution of the drug or therapeutic agent under stirring/shaking, during which the drug loading is highly driven by the concentration gradient, the competition between drug (adsorbate) and MSNs (adsorbent), adsorbate and solvent, and adsorbent and solvent [177,178]. As such, a loading process has been reported with a variety of drugs, such as camptothecin (hydrophobic anticancer molecule) [90], doxorubicin (Dox) hydrochloride [179], curcumin [69], quercetin [68], 5-fluorouracil (5-FU) [180], erythromycin [181], alendronate [182], silymarin [183], and paclitaxel (PTX) [184]. Importantly, the degree of drug loading can be maximized by choosing the desired solvent for the drug, modifying the MSN surface, and adjusting the loading parameters (e.g., time, temperature) [10,86,185].

In the second strategy with capping as the “gatekeeper” for the pore openings of MSNs [168], the first stage is to engineer the outer surface of MSNs via many techniques: molecular or supramolecular functionalization, capping with nanoparticles, and coating with polymer, protein, or lipid. This approach can control the release and delivery of therapeutic agents. In the molecular or supramolecular approach, caps are mainly rotaxanes, pseudorotaxanes, and others consisting of a long chain-like molecule that is threaded via a cyclic molecule [186]. Under certain conditions (e.g., pH, redox), the cyclic molecule can attract rotaxane (to one end of it), with the presence of a stimulus allowing it to slide to the other end. By attaching the thread near the pore opening on MSNs, the sliding cyclic molecule blocks the pore when it is near the particle or opens if it slides away. The idea of nanoparticles as gatekeepers was pioneered by Lin and co-workers [187,188,189,190] with many nanoparticles, such as iron oxide nanoparticles and gold nanoparticles. These small nanoparticles can graft on top of MSNs loaded with cargos through chemical bonding upon cleavage of the chemical bonds linking the nanoparticles with MSNs. Consequently, under certain conditions (pH, redox), external stimuli can trigger the release of cargos in a controlled manner. Next, in the coating strategy, different types of biomaterials, such as polymer, proteins, and lipids, can be introduced onto the surface of MSNs loaded with drugs. Drug release can occur upon degradation of these biomaterials or changing the surrounding environment stimuli, either external or internal [191,192,193]. Table 5 lists some examples of reported studies on prodrug loading in MSNs and their loading capacity. Table 6 provides the different loading strategies and their relationship to stimulate release under various conditions for MSNs, showing the connection between loading and release effects.

### 3.2. Drug Delivery Strategies

**Figure 14:**
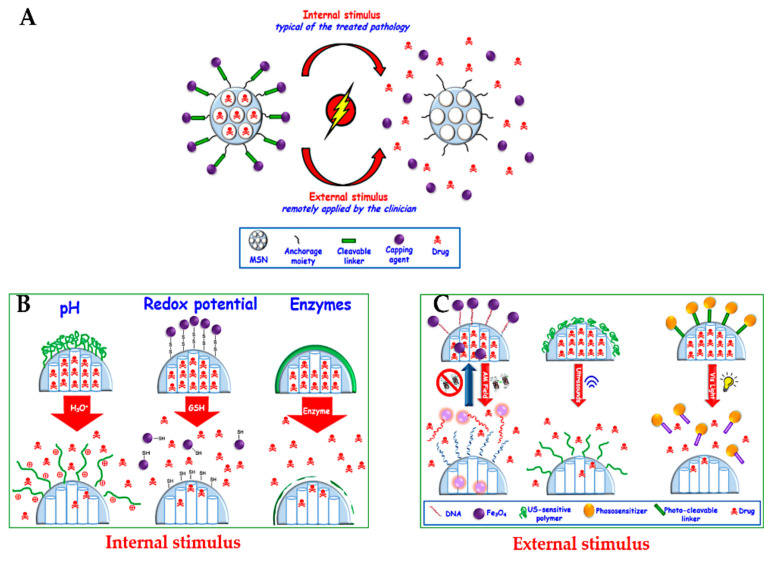
(**A**) Schematic representation of stimuli-responsive release of drugs from MSNs. (**B**) Internal stimuli-responsive release. (**C**) External stimuli-responsive release. Reproduced from [81], MDPI, 2017.

In this section, we provide a summary of delivery strategies that have been developed to treat cancer. This topic is well discussed in several reports for MSN delivery systems, and the readers are referred to these selected reviews [15,32,79,168,218,219,220,221]. Open pores on MSNs, the so-called cavities due to their porous structure, are not only used to load therapeutic agents, but also allow them to diffuse out in the surrounding solution. Closing these pores loaded with therapeutic agents is an essential step to avoid their premature release into the blood vessels, protecting from several side effects because of non-specific release [221]. Much effort has been made in controlled delivery systems with the stimulated or responsive release of therapeutic agents under certain conditions. Two major common strategies for delivering drugs have been reported by internal stimuli release (typical of the treated pathology), such as pH, redox potential, and enzymes, or by external stimuli (remotely applied by the clinician), such as magnetic fields, ultrasound, and light (Figure 14) [32].

#### 3.2.1. Internal Stimuli-Responsive Drug Release from MSNs

##### pH-Responsive Release

**Figure 15:**
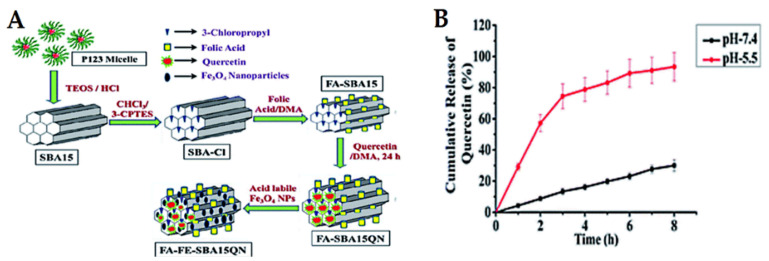
(**A**) Schematic representation of the delivery design for quercetin “FA-FE-SBA15QN”. (**B**) The release kinetics of quercetin from FA-FE-SBA15QN at different pH (7.4 and 5.5). The values are represented as the mean ± SEM. Reproduced with permission from [223], The Royal Society of Chemistry, 2020. This article is licensed under a Creative Commons Attribution-Non-commercial 3.0 Unported License.

Cancer is well-known for its acidic tumor microenvironment with a lower pH than healthy cells/tissues. Consequently, pH-sensitive release is one of the approaches used in cancer nanomedicines. The most investigated pH-responsive delivery systems for anticancer therapeutic drugs have been inspired by applying diverse techniques and vary according to the loading strategies. In this section, we focus on some examples of recent studies published for natural anticancer prodrugs with pH-sensitive release. Nasab et al. [222] fabricated MSNs (MCM-41) capped with chitosan polymer and subsequently loaded with curcumin. This pH-responsive design depends on the degradation of chitosan, allowing high curcumin release at a low pH of 5.5 and resulting in low release at normal physiological pH (7.4). This is favorable for killing U87MG glioblastoma cancer cells. Mishra et al. [223] synthesized MSNs (SBA-15), followed by folic acid functionalization and further loading with quercetin and acid-labile magnetic nanoparticles (Figure 15). The system was investigated in vitro and in vivo in HCT-116 human colorectal carcinoma cells. The results showed that quercetin release was a pH-dependent effect, increasing with decreasing pH. Eventually, the system exhibits promising chemo-theranostic effects for managing colon carcinoma. In this context, Rashidi et al. [224] reported that the release of gallic acid (GA) from MSNs strongly depends on the pH levels of the release media. Furthermore, a pH-sensitive delivery system for ursolic acid prodrug was synthesized by incorporating an acid-sensitive linkage between the drug and MSNs [200]. This sustained release of ursolic acid enhances the anticancer effects against hepatocellular carcinoma cancer. A pH-responsive release of evodiamine and berberine was also achieved by loading them into lipid-coated MSNs [225]. In another strategy using Fe3O4 nanoparticles as gatekeepers, artemisinin is initially loaded into the inner space of hollow MSNs and Fe3O4 capped onto the pore outlets through acid-labile acetal linkers. The results proved that the system is stable under neutral conditions at pH 7.4 (no release), but it releases the prodrug upon exposure to the acidic lysosomal compartment (pH 3.8–5.0). The acetal linkers can be hydrolyzed under acidic conditions. This delivery system has an efficient and desirable anticancer action [226].

##### Redox-Responsive Release 

**Figure 16:**
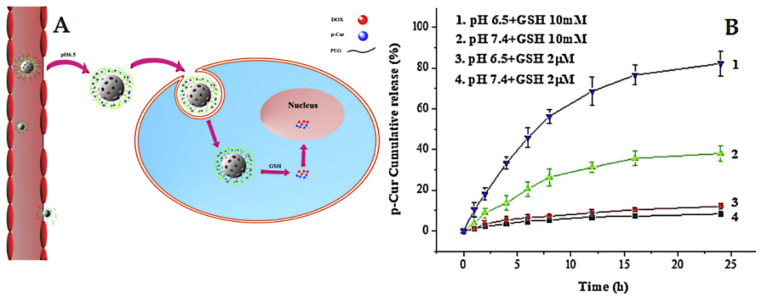
(**A**) Illustration of the dual-response release of p-Cur and Dox co-delivery. (**B**) In vitro release profiles of Cur from MSN/SP/bPEG at 37 °C. Error bars indicate standard deviation. Reproduced with permission from [228], Elsevier B.V, 2019.

The delivery systems that consider redox-sensitive release are popular in cancer-targeted therapy. They take advantage of intracellular conditions and rely specifically on the presence of glutathione (GSH) with a high level of expression in cancer cells compared to normal cells [227]. For example, Lin et al. [228] prepared pH and redox dual-stage responsive release of curcumin with Dox through specific cleavable PEGylation and hydrogel coating (crosslinked by disulfide bonds). The used MSNs were loaded with Dox, whereas the curcumin was encapsulated in a hydrogel coating. The results indicated that dual-responsive release by means of GSH and pH allows efficient and specific cancer targeting (Figure 16). In another example, Xu et al. [229] developed a stimuli-responsive delivery for curcumin gatekeepers based on MSNs characterized by large pores (named LP). In this design, curcumin is anchored to the surface of LP using thiol-ene as the click chemistry approach, followed by a coating of the pluronic polymer (F127) on the surface by means of self-assembly. The release studies proved that curcumin exhibits a redox-responsive release depending on the absence or presence of GSH at different pH levels. 

##### Enzyme-Responsive Release

In the human body, many chemicals and enzymes are inherently expressed during pathological conditions, including cancers, which are explored to trigger drug release from numerous MSN types [10]. A delivery system tailored for anticancer treatment with enzyme-responsive release, in which matrix metalloproteinase (MMP) substrate peptide containing PLGLAR, which is sensitive to MMPs, is immobilized onto amine-modified MSNs and further capped with bovine serum albumin by covalent bonding. The results revealed that the nanoplatform delivery exhibits enzyme-triggered release of drug and efficiently inhibits tumor growth in vivo. MMP enzyme-trigger release of cisplatin-based MSNs was reported by Vaghasiya [230]. The system constructed by coating collagen on the surface of drug-loaded MSNs eventually results in sensitive enzyme release. 

#### 3.2.2. External Stimuli-Responsive Drug Release from MSNs

##### Responsive Release Using Magnetic Fields

This approach is largely employed for responsive release due to the magnetic guidance by a permanent magnetic field and locally increases the internal temperature by changing the magnetic field potential [32]. The delivery systems concerning this method widely use magnetic nanoparticles (superparamagnetic iron oxide-SPIONs) 5–10 nm in size as a core and mesoporous silica shell permitting drug loading and release [231]. Regarding natural prodrugs, the nano platform developed by Janus MSNs consists of magnetic nanoparticles to achieve magnetic targeting and delivery of berberine. This system produces a sustained release and exerts extraordinarily site-specific internalization into hepatocellular carcinoma cells, facilitating a high antitumor effect against liver cancer due to an external magnetic field [232]. Another very recent example is Asgari et al. [233] developing a novel in situ encapsulation delivery for curcumin consisting of magnetite-silica core-shell nanocomposites. The system could be effective for clinical application by means of magnetic hyperthermia therapy. In addition, nanoparticles of DNA-capped magnetic mesoporous silica composite exhibit temperature-dependent release of Dox and magnetic hyperthermia effects against cancer [234].

##### Responsive Release of Drugs Using Light

As a non-invasive and spatiotemporal strategy, different wavelengths of ultraviolet, visible, or near-infrared light can be employed to trigger and control drugs from MSNs. The main advantages are easy application by the clinician and focalization to the target tissue [235,236,237]. 

**Figure 17:**
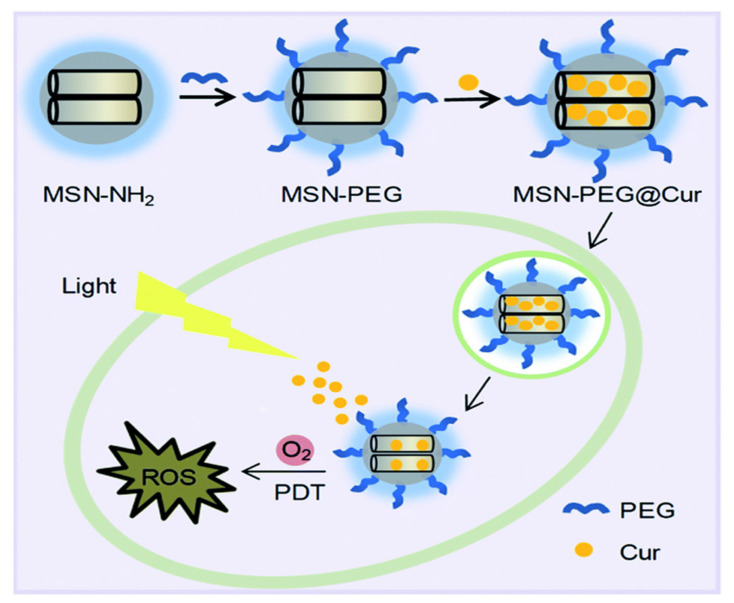
The preparation process for MSN-PEG@Cur and schematic representation of the intracellular photodynamic therapy (PDT) process after endocytosis of MSN-PEG@Cur. Reproduced with permission from [238], The Royal Society of Chemistry, 2020. This article is licensed under a Creative Commons Attribution-Non-commercial 3.0 Unported License.

Kuang et al. [238] developed a curcumin delivery system by means of photodynamic therapy, achieving PEGylated MSNs loaded with curcumin (Figure 17). The results demonstrated that the developed system, “MSN-PEG@Cur”, exhibits efficient endocytosis into cells and the release of curcumin. As a photodynamic therapy, it promptly generates ROS upon irradiation, allowing effective treatment for cancer. In another example, Li et al. [239] preloaded berberine into folic acid-modified Janus gold MSNs. The in vitro and in vivo results demonstrated that the delivery system verifies sustained release dependent on light and an efficient anti-tumor effect with good biosafety for normal tissue. Feng et al. [225] fabricated a dual delivery platform for evodiamine and berberine loaded into lipid-coated MSNs with thermo-sensitive release. Their results suggest that the temperature-responsive release is promising for both hydrophobic and hydrophilic drugs. Using an important natural prodrug of capsaicin, the main ingredient in red or hot chili pepper, Yu et al. [240] reported a novel design of NIR-triggered plasmonic nanodot-capped MSNs for inhibiting metastasis of human papillary thyroid carcinoma. The nanoplatform consisting of gold nanodot-capped MSNs loaded the prodrug. The results depicted that the delivery of capsaicin by the developed nanoformulation exhibited strong cytotoxicity against the FTC-133 and B-CPAP cell lines compared to free capsaicin. 

##### Responsive Release of Drugs by Ultrasound

Ultrasound is considered an interesting and efficient approach to trigger the release of drugs from MSNs. The main advantages include deep penetration of living tissues without causing damage, and it is non-invasive and can be concentrated to the desired tissue [32,241]. In this approach, drugs can be released from pores of MSNs due to the thermal effect of ultrasound radiation on chemical bonds and thermosensitive polymers while closing in the absence of a radiation effect [242,243,244]. An example is MSNs modified with amine groups covered by sodium alginate polymer and subsequently loaded with a model cargo (rhodamine B) [245]. The results indicated that rhodamine B releases based on changing the ultrasound potential (ultrasound on–off responsiveness).

## 4. Selective Targeting Strategies for Cancer

One of the hottest areas in delivery systems is the delivery of drugs or therapeutic agents directly to specific tissues where the desired therapy is required. The main goal of nanomedicine application for cancers is avoiding the expected side effects from drugs and damaging the healthy cells surrounding the tumor site [21,246]. Two routes have been used depending on nano-particulate delivery for cancers, passive and active selective targeting. 

**Figure 18:**
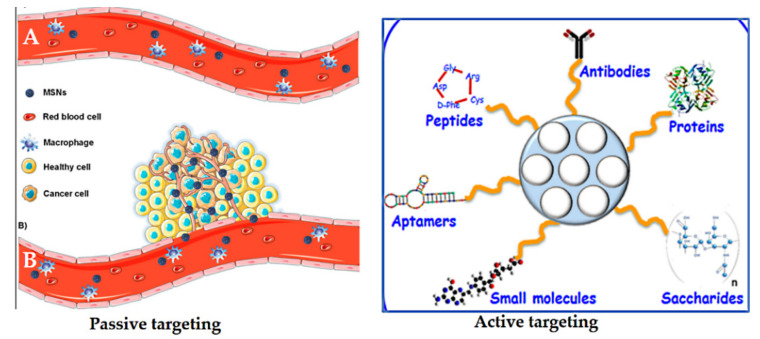
Schematic representation of the enhanced permeability and retention (EPR) effect (lift side). (**A**) Normal blood vessels (no fenestrations), showing that MSNs remain in the bloodstream. (**B**) Tumor tissues (defective blood vessels present) showing that MSNs leak out through the endothelial gap–gap and eventually accumulate in the tumor. On the right is a schematic depiction of active targeting with a variety of possibilities depending on the MSNs. Reproduced from [32,259], MDPI, 2020.

Passive targeting was first postulated by Matsumura and Maeda in 1986 [247]. Nanoparticles can accumulate in tumor tissue by the enhanced permeability and retention (EPR) effect. They hypothesized that the localization of macromolecules and particles of certain sizes differ, which is attributed to the tumor microenvironment, the relatively slow elimination rate, and poor lymphatic drainage. Particle size, surface charge, or hydrophobicity can be mediated by the so-called EPR effect, or passive targeting [248,249] (Figure 18). Passive targeting is due to abnormalities in tumor blood vessels, which have wide interendothelial junctions with pores (700 nm). Injected nanoparticles travel through the bloodstream and accumulate in the tumor interstitium because of this characteristic of tumor vessels [247,249]. The nanoparticles already located in the tumor would remain there because of the ineffective lymphatic drainage with the fast growth of the tumor tissue [221]. However, the EPR effect is often not efficient enough to selectively deliver and reduce the side effects of anticancer drugs [250]. 

Active targeting is used to enhance the ability of a nanoparticle delivery platform carrying drugs to be taken up and bind to cancer cells via specific receptors on their surfaces compared to normal cells [251]. It is well known that some tumor cells overexpress certain receptors on their surface. Thus, nano-delivery systems functionalized with various ligands permit a high affinity for receptors facilitating specific retention and uptake by cancer cells. Thus, the role of targeting ligands is to allow the nanocarriers to selectively enter the cancerous cells, but not normal cells. This not only reduces the administration dosage, but also diminishes toxic side effects of drugs during circulation [252]. Many ligands have been investigated to functionalize/decorate nano-delivery systems based on MSNs for selectively targeting cancers (Figure 18). These include antibodies, proteins, peptides, aptamers, small molecules, and saccharides [221]. For example, transferrin [237], folic acid [42], epidermal growth factor (EGF) [253], methotrexate [254], RGD-type peptide [255], anti-HER2/neu [256], hyaluronic acid [257], and mannose [258]. 

**Figure 19:**
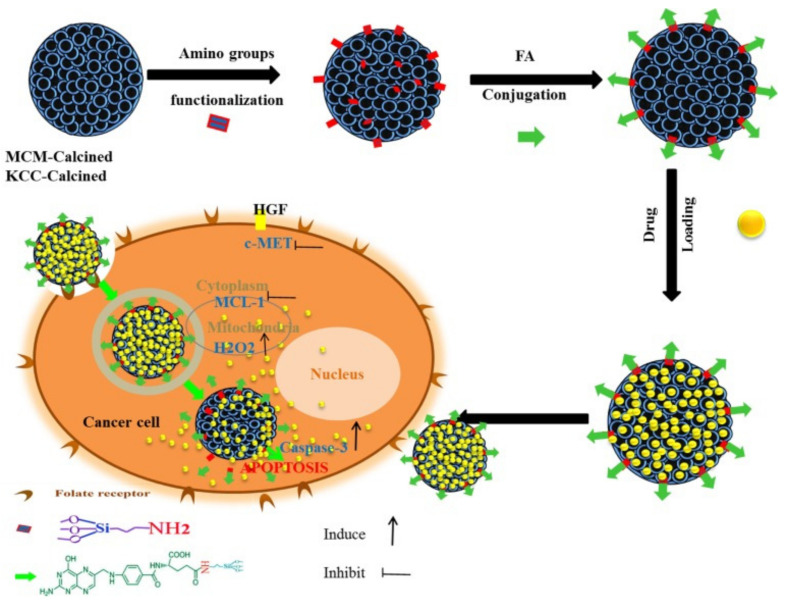
Schematic representation of the preparation, internalization, and anticancer mechanism of action of the prepared nanosystem in human liver carcinoma (HepG2) cells. This schematic shows the prodrug release into cancer cells and the main anticancer action for inducing apoptosis via activation of caspase-3 for killing HepG2 cancer cells proposed by assistance from important signaling pathways (c-MET, MCL-1, and H2O2). Reproduced from [42], Impact Journals, 2018.

As an example, Kundu et al. [203] designed targeted delivery for umbelliferone prodrug, with the system consisting of umbelliferone loaded in MSNs and capped with a pH-sensitive poly acrylic acid and further grafted with folic acid on the surface. The delivery with folic acid conjugation increases the anticancer potential of umbelliferone against breast cancer cells. In another example, Yinxue et al. [199] investigated myricetin prodrug (Myr)-loaded MSNs combined with multidrug resistance protein (MRP-1) siRNA and the surface modified with folic acid to treat non-small cell lung cancer (NSCLC). In vivo fluorescence demonstrated that folic acid-conjugated MSNs with Myr and MRP-1 nanoparticles could specifically accumulate at tumor sites. Compared to free Myr and MSNs combined with MRP-1/Myr nanoparticles, folic acid-conjugated MSNs loaded with Myr and MRP-1 nanoparticles could more effectively suppress tumor growth with few side effects. Overall, a folic acid-conjugated nanoparticle system could provide a novel and effective platform for the treatment of NSCLC. We also reported a targeted delivery system consisting of folic acid conjugated to amine-modified MSNs (KCC-1 and MCM-41) and subsequently loaded with various prodrugs (curcumin, colchicine, and quercetin) [42]. The nanoformulation containing curcumin exhibited the highest anticancer activity against liver cancer cells through apoptosis via caspase-3, H2O2, c-MET, and MCL-1 (Figure 19). Table 7 lists some other examples of targeted delivery systems for anticancer natural prodrugs.

## 5. Motivation towards Natural Anticancer Agents

Nature is a great source of thousands of chemical substances/compounds generally considered natural products, as well as natural prodrugs if they are used for treating diseases [269,270]. Natural products (of natural origin) and herbal medicines have been used in traditional and modern medicine to treat cancer, and account for nearly 60% of pharmaceutical drugs [271,272,273,274,275,276,277,278,279]. Natural prodrugs provide medical effects against cancers as either chemotherapeutics or chemopreventive drugs. Regarding chemotherapeutics, anticancer natural prodrugs have been utilized for various cancer treatments and are becoming rising stars in the field of drug discovery for their contributions [280]. Some available drugs used in clinical applications for cancer patients diagnosed with different cancers are derived from plants, including vincristine, vinblastine, topotecan, and taxol [281]. There are also some examples of anticancer drugs originating from microbes, including Dox, daunorubicin, and bleomycin. Regarding cancer prevention, there are numerous natural substances (e.g., in fruits and vegetables) that have also been applied in cancer prevention along with human health enhancement with no detectable side effects [282]. To achieve cancer prevention goals, by completely preventing or delaying cancer, the main strategies that can be used, such as maintenance (healthy lifestyle), avoidance (exposure to toxicants/carcinogens), and dietary consumption (chemopreventive substances to drugs) [283]. There is no doubt that prevention leads to better management and treatment of tumor growth and the risk for developing metastases, secondary tumors, and recurrence [283]. Eliminating cancer, decreasing metastasis, reducing reappearance, and improving patient survival are key to curing cancers [284].

**Figure 20:**
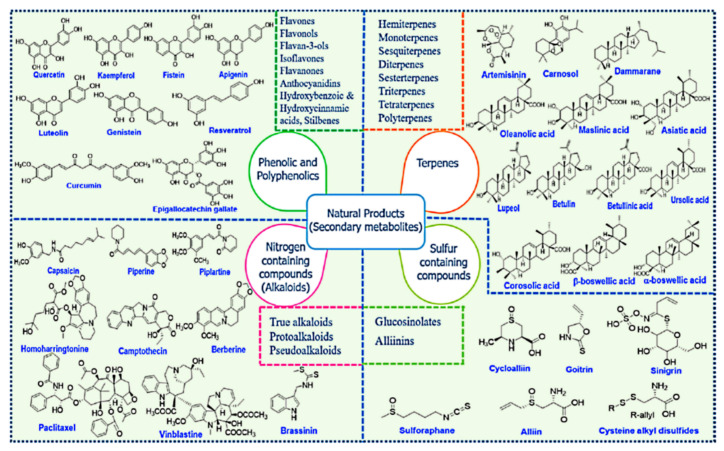
Chemical structures of various classes of natural compounds (prodrugs). Reproduced with permission from [285], Elsevier Ltd., 2019.

Among the main natural sources, plants are a considerable domain for supplying a variety of natural products with diverse chemical structures with a wide range of health benefits. The natural products are the main secondary metabolites produced by plants and can be classified into four major classes: phenolics and polyphenolics, terpenes, nitrogen-containing alkaloids, and sulfur-containing compounds (Figure 20) [285,286,287].

In recent years, attention has been focused on solving the problems associated with natural prodrug substances to increase their use in cancers and other pathological disorders. As an advanced strategy, nanotechnology application in medicine, called nanomedicine, is a promising approach being developed to overcome the limitations of natural prodrugs and improve their efficiency in cancer therapy. The advent of nanomedicine for cancer therapy occurred recently, and the rate of its progress and transformation in cancer treatments has also been rapid [285]. This technology can solve the major drawbacks of natural anticancer prodrugs, including low aqueous solubility, low bioavailability, multidrug resistance, and non-specific targeting. The developed nanoformulations for delivery of natural anticancer prodrugs are intentionally being explored with several classes of prodrugs based on various organic and inorganic nanocarriers [285,288,289,290,291,292,293,294,295,296,297,298]. By reviewing in vitro and in vivo cancer models in the literature, it seems that nanoplatforms for delivering anticancer natural prodrugs have potentially improved the therapeutic activity, specific targeting, solubility, and bioavailability, and reduced side effects. The better patient response and survival are accompanied by possible enhancement of the pharmacological impacts and clinical outcome. Below, we discuss the delivery systems that have been established for select anticancer natural prodrugs employing MSNs. 

### 5.1. Curcumin

Curcumin (1,7-bis(4-hydroxy-3-methoxyphenyl)-1,6-heptadiene-3,5-dione) is a natural hydrophobic polyphenol compound, and is the major constituent derived from turmeric rhizome (*Curcuma longa* L.). Turmeric is a well-known spice in the kitchen and has a long history in traditional medicine for a wide range of diseases. Curcumin has numerous pharmacological activities, including anticancer, antiviral, antioxidant, anti-inflammatory, wound healing, and antimicrobial, among others [299,300,301,302,303,304,305,306,307,308,309,310,311,312,313,314,315].

Despite these potential pharmacological activities, the pharmacokinetics of curcumin show inherently poor solubility and bioavailability because of the limited absorption, rapid metabolism, and quick systematic elimination [316,317,318,319]. To take advantage of the medical actions of curcumin and improve the inherent limitations, versatile nanoplatform delivery systems have been constructed and studied, including MSNs. Concerning MSNs for curcumin delivery contribution, MSN-based nanosystems show great promise for combating cancers and will be seen soon in clinical stages. 

Ma’mani and co-workers [196] fabricated guanidine-functionalized PEGylated KIT-6 MSNs 60–70 nm in size for delivery of curcumin to breast cancer cells. The system exhibited pH-sustained release of curcumin with long-term anticancer efficacy in human breast cancer cells (MCF-7 human breast adenocarcinoma cells, 4T1 mouse breast cancer cells, and MCF10A human mammary epithelial cells). A similar trend was observed for MSNs, namely MSU-2 and MCM-41 loaded with curcumin showing significant anticancer effects against different cancer cells (A549 human lung carcinoma cells, MCF-7 human breast cancer cells, and B16F10 mouse melanoma cells) compared to pure curcumin [320]. In further investigations, they found that the plausible mechanism contributing to anticancer effects is the generation of intracellular ROS and the induction of apoptosis. Lin et al. [228] tailored a co-delivery system of Dox loaded into MSNs as the core and curcumin loaded into the polymeric coating shell. The results indicate the long duration of blood circulation due to the PEG shell, GSH-sensitive release effect for drugs, and high cellular uptake resulting in synergistic anticancer effects through enhanced apoptosis of Hela cells. As an interesting nanoplatform, the fabricated lipid bilayer-coated curcumin-based MSNs unveiled a controllable and highly biocompatible theranostic nanosystem for cancer delivery [321]. Another recent strategy for building a delivery system for curcumin is by loading the prodrug into amino-MSNs using APTES silanes (KIL-2 and KIT-6), then coated by polyelectrolyte polymer complex by means of the layer-by-layer technique [197]. Based on the comparative data from this study, the nanoformulation exerts an anticancer effect on human cell lines, namely HL-60, EJ, and HEK-293, compared to free curcumin, demonstrating the promising delivery of prodrug with a sustained release effect. Considering active cancer-targeting designs, our group constructed selective targeted anticancer delivery of curcumin using MSNs (KCC-1-NH2-FA-CUR and MCM-41-NH2-FA-CUR) showing selective targeting of liver cancer cells (HepG2). The killing mechanism was found to be apoptosis [42]. The aspartic acid-functionalized PEGylated MSN-graphene oxide loaded with curcumin exhibited pH-sensitive release and excellent killing of breast cancer cells (MCF-7) [322]. With the occurrence of drug resistance in come cancers, silver-decorated SBA-15 (as metal-doped nanocomposites) coated with melanin-like polydopamine was used to deliver curcumin [323]. They found that the utilization of a nanoplatform containing curcumin enhances anticancer efficiency against select cancer cells (HeLa and taxol-resistant NSCLC (A549/TAX) compared to free curcumin.

To verify the antitumor action against breast cancer in vivo, Gao et al. investigated PEGylated lipid bilayer-coated MSNs for a dual-delivery of PTX and curcumin with prolonged release to determine their pharmacokinetic properties, uptake, subcellular localization, biodistribution and tumor site targeting, and effectiveness [324]. The delivery system could significantly increase the anti-tumor effect either by intravenous or intratumoral administration compared to free drug. The nanoplatform effectively led to the accumulation of the nanoformulation carrying drugs in the tumor site, resulting in highly efficient therapeutic effects in breast cancer. As such evidence of utilization of curcumin for co-delivery systems is important for further improvements and reducing side effects and drug resistance in cancers, which is the main issue for conventional cancer therapy. Sun et al. [325] conducted a study of cancer targeting by means of folic acid and PEI-modified-MSNs for curcumin; they concluded that the system exhibits sustained release (pH-sensitive delivery), which is suitable for antineoplastic drugs. Several studies have reported the delivery of curcumin in different cancers in vitro or in vivo (Table 8).

### 5.2. Quercetin

Quercetin is a dietary flavonoid compound derived from plants (e.g., medicinal plants, vegetable, fruits). It is a 3,3′,4′,5,7-pentahydroxyflvanone named by the International Union of Pure and Applied Chemistry (IUPAC) [337]. Quercetin has unique biological properties that play an important role in mental/physical performance, as well as reducing infection risk [338]. It has shown numerous pharmacological actions, including anti-oxidant, anti-microbial, anti-diabetic, anti-inflammatory, anti-cancer, anti-Alzheimer, psychostimulant, mitochondrial biogenesis stimulant, lipid peroxidation inhibitor, platelet aggregation inhibitor, and capillary permeability inhibitor, among others [339,340,341,342,343,344,345,346,347,348]. The dietary intake of quercetin varies in many countries. The estimated intake dosage of flavonoid (quercetin accounts for nearly 75%) ranges from 50–800 mg/day according to the consumption of fruits, vegetables, tea, and herbals [349]. In addition, quercetin is safe with a single dose of up to 4000 mg orally and up to 100 mg via intravenous administration [350]. Quercetin is an excellent free radical scavenging antioxidant [344] and is considered one of the most effective antioxidants [351]. Consequently, quercetin exhibits promising effects against cancer [339,352] in vitro and in vivo [353,354,355,356,357,358,359,360]. Nevertheless, its potential impacts in clinical applications are drastically limited due to its poor solubility, low bioavailability, and instability [361]. According to the pharmacokinetics of quercetin in humans, only ~2% is bioavailable (from single dose) with an absorption rate of 3 to 17% (from 100 mg applied in individual healthy persons) [337]. The factors affecting oral bioavailability are low absorption, extensive metabolism, and/or rapid elimination, in addition to low solubility and non-selective targeting of cancers. Several nanoplatform delivery systems focus on overcoming these challenges to introduce quercetin into clinical applications soon for cancer [362,363,364,365,366,367,368].

The use of MSNs to develop new delivery systems for quercetin against cancers has attracted many research groups. Liu et al. [369] fabricated a system for dual delivery of PTX and quercetin into MSNs to overcome multidrug resistance in breast cancer. The nanosystem exhibited CD44 receptor-mediated active targeting for MCF-7/ADR cells. At the same time, the addition of quercetin with PTX significantly improves the sensitivity of MCF-7/ADR cells to PTX, providing a solution to multidrug resistance in breast cancer. Huang et al. [370] designed a novel nanoformulation consisting of quercetin-loaded MSNs coating cancer cell membranes for enhanced tumor targeting and radiotherapy. In vitro and in vivo investigations revealed that the system has many advantages, including excellent tumor targeting ability and efficient inhibition of tumor growth. The platform fulfills innovative ideas for targeting cancer and improving therapy. In another attempt, polydopamine-coated hollow MSNs combining Dox hydrochloride with quercetin efficiently overcame multidrug resistance in taxol and Dox double-resistant human colorectal cancer cells (HCT-8/TAX cells) [371]. Fang et al. [262] developed a hyaluronic acid-modified MSNs that co-deliver quercetin and Dox to enhance the efficacy of chemotherapy for gastric carcinoma. They found that the system enables stability, sustained release, and selective killing effects. An in vivo study disclosed that the co-delivery significantly enhances the anticancer efficacy compared to a single drug, showing the importance of quercetin in clinical application. In this context, Murugan et al. [264] loaded topotecan into the pores of MSNs, followed by poly(acrylic acid)-chitosan as an outer layer to further conjugate quercetin, and then grafting with arginine-glycine-aspartic acid (cRGD) peptide on the surface as targeting ligands for cancers. The system released the drugs as a function of pH and uptake occurred through integrin receptor-mediated endocytosis, enabling efficient anti-tumor effects in multidrug resistant breast cancer cells and animal studies. As far as active targeting and bioavailability are concerned, MSNs conjugated with folic acid and loaded with quercetin exhibit higher cellular uptake and more quercetin bioavailability in breast cancer cells, as well as an enhanced antitumor effect through apoptosis [265]. These studies demonstrate the prospective application of quercetin in cancers by means of single or co-delivery, facilitating efficient targeting and antitumor effects, creating new possibilities for clinical applications.

### 5.3. Resveratrol

Resveratrol (RSV, 3,5,4′-trihydroxy-trans-stilbene) is a natural polystilbene and non-flavonoid polyphenol. As a phytoestrogen compound, RSV is present in a wide range of plants and is abundant in extracts from the grape skin and other fruits and vegetables. RSV has been reported to exert multiple pharmacological effects, including anti-inflammatory, anti-viral, anti-microbial, anti-Alzheimer, anticancer, cardioprotective, neuroprotective, and immunomodulatory actions [372,373,374,375,376,377,378,379,380,381,382,383,384,385,386]. Concerning the anticancer effects on the preclinical level, RSV has also been reported to possess important antitumor actions in several preclinical animal models [387,388,389,390,391,392,393,394,395,396,397,398]. The clinical prospective of RSV has also been evaluated in a few clinical trials. The first clinical trial by Nguyen et al. [399] indicated that the freeze-dried grape powder (containing RSV) effectively inhibits colon cancer in patients. In addition, Patel et al. [374] showed that a daily dose of RSV at 0.5 or 1.0 g produces sufficient anticarcinogenic effects in colorectal cancer. Furthermore, Howells et al. [400] demonstrated that RSV given at micronized formulation with 5.0 g daily for 14 days in patients with colorectal cancer and hepatic metastases prevented malignancies by increasing apoptosis.

Despite promising preclinical (in vitro and in vivo) and prospective clinical results as an anticancer agent, RSV still has many challenges due to the pharmacokinetics, metabolism, bioavailability, and toxicity in cancer patients [374,401]. These associated properties prevent translation into more clinical trials and human benefits. In addition, RSV has shown poor bioavailability due to its quick extensive metabolism, and large doses (up to 5 g/day) should be applied to provide anticancer therapeutic activity [402]. Such high doses result in adverse effects (e.g., diarrhea, nausea, and abdominal pain with >1 g/day) [402]. As the poor bioavailability limits the RSV activity, there are various approaches for overwhelming the bioavailability, including co-delivery with piperine prodrug [403], micronized powders [403], and nanoplatform delivery [404,405,406,407]. Application of nanomedicine can improve the stability and bioavailability, and minimize side effects of RSV, which is making RSV a prospective candidate for treating many diseases, including cancers.

Few investigations in recent years have used MSNs for the delivery of RSV. Chaudhary et al. [408] loaded RSV into MSN-modified phosphonate or MSN-modified amine to improve the anti-proliferative activity and sensitization of drug-resistant prostate cancer. The RSV is released as a function of pH, and the phosphonate-modified nanoparticles effectively kill cancer cells better than amine-modified nanoparticles. Hu et al. [267] constructed a dual delivery system for anti-miR21 and RSV using MSNs conjugated with hyaluronic acid to target gastric carcinoma through overexpression of the CD44 receptor on cell membranes. They found that this nanoformulation has a superior anticancer effect due to synergistic effects specifically delivered by combining anti-miR21 and RSV in gastric cancer cells. Furthermore, Summerlin et al. [409] encapsulated RSV in colloidal MCM-48 and found that the nanoformulation enhances saturated solubility (∼95%) and release effect compared to pure RSV. The nanoformulation also possesses a higher killing ability for HT-29 and LS147T colon cancer cells compared to pure RSV by mediating the PARP and cIAP1 pathways.

### 5.4. Berberine

Berberine is an isoquinoline alkaloid found in a handful of plants widely used in botanical medical practice, such as *Hydrastis canadensis* (Goldenseal), *Berberis aquifolium* (Oregon grape), *Berberis vulgaris* (Barberry), and *Coptis chinensis* (Chinese Goldthread) [410,411]. Versatile pharmacological activities have been reported for berberine, including anti-viral, anti-microbial, anticancer, anti-diabetic, anti-diarrhea, and anti-inflammatory, and treatment for congestive heart failure, cardiac arrhythmia, and hypertension. Recently, berberine extract or pure compound has gained much attention in the newly published research and is among the top pharmaceutical supplements on shelves [412]. The preclinical evidence from huge studies reveals the capability of berberine to treat many diseases [411,413,414,415,416,417,418,419]. Thus, berberine is clinically studied for many diseases, such as diabetes [410,420,421,422,423]. Particular attention has been given to berberine in cancers, so it is expected to be one of the most common natural compounds under the scope of extensive clinical investigations of cancers [424]. The main challenges in translating berberine to the clinical application are low solubility, poor aqueous solubility, slight absorption, and low bioavailability. There are some strategies to deal with these limitations, such as producing berberine hydrochloride to increase its solubility. Another approach is encapsulating berberine into nanocarriers for nanoplatform delivery [425,426,427].

Berberine loaded into folic acid-conjugated gold-MSNs shows superb anticancer effects, good biosafety, and protection of normal tissue in vitro and in vivo for chemo-radiotherapy of liver cancer [239]. Another conformation obtained by Feng et al. [225] showed that MSNs based on dual delivery of hydrophobic prodrugs with berberine and evodiamine through thermo/pH-responsiveness improves antitumor effects in vitro and in vivo. Other results propose that the berberine-loaded Janus nanocarriers (MSNs containing iron oxide) driven by a magnetic field provide an effective and safe approach against hepatocellular carcinoma [232]. As with other drugs, berberine can be released depending on different conditions; by disulfide bond linking, berberine releases from MSNs under glutathione conditions upon breakage of the disulfide bond, promoting the anticancer action against liver cancer [428].

### 5.5. Thymoquinone

Thymoquinone (TQ, 2-methyl-5-isopropyl-1,4-benzoquinone), a monoterpene diketone compound, is the main active component in essential oil (volatile oil) of *Nigella sativa* L. (known as black seed or black cumin). TQ was isolated for the first time in 1963 [429] and exhibits various pharmacological activities in vitro and in preclinical investigations. The most reported activities are anticancer, antioxidant, anti-microbial, neuroprotective, anti-inflammatory, anti-microbial, and anti-diabetic [430,431,432,433,434,435,436,437,438,439,440]. A considerable amount of available data from preclinical studies encourage the translation of TQ into clinical settings. There is no doubt of the promising anticancer effects of TQ, but the lack of bioavailability and pharmacokinetic parameters delay the use of TQ in clinical applications. The main issues are low bioavailability, solubility, biodistribution in the body, rapid metabolism, and excretion [441,442,443]. In recent years, several strategies have been investigated to improve these limitations, such as the development of novel analogs [444], use of different routes (e.g., oral, intraperitoneal, intravenous), and nano-delivery systems [296,445,446].

Few delivery systems have been designed for TQ based on MSNs. The TQ-loaded MSNs produce more anticancer effects against MCF-7 and HeLa cells than pure TQ [447]. In addition, both TQ-loaded MSNs and pure TQ exert their anticancer activity by means of ROS-mediated apoptosis. To enhance the targeting ability towards glioma cells, we fabricated core–shell nanoformulations [44], with the core consisting of MSNs loaded with TQ and the shell consisting of whey protein–Arabic gum or chitosan–stearic acid complex. Interestingly, TQ releases as a function of pH and induces selective killing of cancer cells compared to normal cells. Furthermore, the core–shell nanoformulations significantly kill glioma cancer cells via apoptosis-mediated pathways due to caspase-3 activation, cytochrome c triggers, and cell cycle arrest at G2/M signaling. In this sense, the efficient anticancer effects against brain cancers can be attributed to the distribution of TQ-loaded MSNs [448]. The study showed that encapsulating TQ in MSNs improves delivery to some brain areas, including the cortex, thalamus, hypothalamus, and midbrain, but reduces its delivery to the cerebellum compared to pure TQ. The results also indicated that neither free TQ nor MSN-TQ reaches the hippocampus. Thus, MSNs potentially target TQ to certain brain areas.

### 5.6. Gallic Acid

GA (3,4,5-trihydroxybenzoic acid) is one of the most abundant phenolic acids present in plants (e.g., fruits and medicinal plants. GA can be isolated from different plants of *Quercus* spp. and has extensive applications in the food and pharmaceutical industries [449]. The therapeutic uses include antimicrobial [450], anticancer, gastrointestinal disease, cardiovascular disease, metabolic disease, neuropsychological disease, and other miscellaneous diseases [449,451,452,453,454,455]. GA has a potential antioxidant action modulated by various signaling pathways (e.g., inflammatory cytokines, and enzymatic and nonenzymatic antioxidants) that lead to its therapeutic effects [453]. However, as with many prodrugs, limitations still exist for clinical use of GA and to confirm its therapeutic outcomes. Several nanostructures have been used to fabricate delivery systems to solve these limitations and achieve effectiveness to translate GA into clinical investigations [456,457,458,459,460]. 

Only a few studies have been reported on MSN nanosystems for GA. MSNs functionalized with amino acid and coated with chitosan exhibit a high loading capacity of ~20–38%, leading to better killing potency of MCF-7cells than pure GA [195]. GA is an unstable molecule under specific pH; by encapsulating it in MSNs, the release of GA can be controlled by media with different pH and released in the presence of higher antioxidant activity [224]. With respect to the anticancer effect, incorporation of GA into MSNs by means of covalent bonding increases its activity against HeLa and KB cells, with a killing efficiency of up to 67% [461]. Thus, GA-loaded MSNs easily internalize into Caco-2 cells, releasing GA to enhance cytotoxic effects against colon cancer [462].

### 5.7. Essential Oils

Among the plant natural prodrugs, the essential oils (also known as volatile oils) have particular importance in many sectors (e.g., pharmaceutical, cosmetic, agricultural, and food) [463,464]. With a long history in many cultures, essential oils can be used for different purposes [465]. For example, Ancient Egyptians used essential oils as early as 4500 BC for cosmetics and ointments [466]. They made a mixture of many herbals containing essential oils (e.g., aniseed, cedar, onion, myrrh, grapes, etc.) as preparations in perfume or medicine. In recent years, the most important use of essential oils has been aromatherapy due to their curative effects [467]. Essential oils are complex mixtures of volatile compounds found especially in aromatic plants, such as clary sage (*Salvia sclarea* L.), eucalyptus (*Eucalyptus globulus* Labill.), geranium (*Pelargonium graveolens* L.), lavender (*Lavandula officinalis* Chaix), lemon (*Citrus limon* L.), peppermint (*Mentha piperita* L.), roman chamomile (*Anthemis nobilis* L.), rosemary (*Rosmarinus officinalis* L.), basil (*Ocimum basilicum*), rosemary (*Rosmarinus officinalis*), and ginger (*Zingiber officinale*). The essential oils are obtained from plant sources by several methods, such as hydrodistillation, steam distillation, cold pressing, solvent extraction, microwave-assisted processing, and carbon dioxide extraction. Concerning their chemical composition, essential oils were originally characterized as monoterpene and sesquiterpene hydrocarbons together with their oxygenated derivatives, besides the aliphatic aldehydes, alcohols, and ester structures [466]. Due to the chemical compositions of essential oils with versatile compounds that possess many roles and modes of action in various pharmacological entities and therapeutics, including anticancer, cardiovascular disease treatment, anti-bacterial, anti-viral, anti-oxidants, analgesics, and antidiabetics [468,469]. The main applications are enhanced transdermal drug delivery due to their skin penetration, and aroma and massage therapy [470]. Essential oil compounds have been reported to have potential anticancer effects in vitro and in animal models [471,472,473,474,475,476,477]. However, essential oils generally have low stability, high volatility, and a high risk of deterioration by exposure to direct heat, humidity, light, or oxygen [478]. Nanoformulations are a recent strategy being developed for essential oils and their constituents to solve these problems [463,479,480,481,482].

To the best of our knowledge, no anticancer nanoformulations have been designed for essential oils and their constituents. MSNs are efficient particles for the high loading of essential oil substances. Melendez-Rodriguez et al. demonstrated that eugenol, an important component in various essential oils of herbs, is efficiently encapsulated in pores of MSNs up to 50 wt.%. by means of vapor adsorption [483]. Ebadollahi et al. [484] reported that the loading of essential oils of thymus species into MCM-41 increases their stability and persistence up to 20 days. Furthermore, Janatova et al. [485] demonstrated that different encapsulated essential oil components in MCM-41 provide long-term effects through controlled release compared to the same pure substances. In addition, Jobdeedamrong et al. [486] showed that the release of essential oils (peppermint, thyme, cinnamon, and clove oil) is controlled by loading them into MSN-functionalized particles and grafting them with hyaluronic acid. Confirmation of delayed volatilization was reported for lavender oil loaded into MSNs [487]. Jin et al. [488] showed that MCM-41 modified nanoparticles enable high loading of pepper fragrant along with bactericidal activities against different microbes. Thus, the incorporation of essential oils from different herbs could be used effectively for infectious diseases [489,490] and treating biofilm [491]. 

### 5.8. Other Natural Products 

Artemisinin is a sesquiterpene lactone derived from *Artemisia annua*. It is used as an antimalarial for treating multi-drug-resistant strains of falciparum malaria. It has also shown promising anticancer effects [492]. Artemisinin loaded into pores of hollow MSNs and capped with Fe3O4 nanoparticles act as gatekeepers [226]. The system shows a pH-dependent release effect, with stable release achieved at pH 7.4 and higher artemisinin release at low pH (3.8–5.0). This system exhibits excellent anticancer efficacy. Another multifunctional nanocarrier, Fe3O4@C/Ag@mSiO2 loaded with a high amount of artemisinin, allows pH-stimuli release and more killing of HeLa cancer cells compared to free artemisinin [493].

Some natural prodrugs are toxic compounds, and this toxicity prevents them from being used to treat cancers. An important example is colchicine, a natural alkaloid derived mainly from *Colchicum automnale*. It has long been used clinically to treat gout and familial Mediterranean fever. Colchicine is an important antimitotic prodrug and efficiently kills cancer cells [494], but the major challenge to its use is its toxicity. Earlier, Cauda et al. reported a one-step fabrication of colchicine-loaded in lipid bilayer-coated MSNs, making the system more stable and leading to effective microtubule depolymerization upon cell uptake [495]. We also loaded colchicine into folic acid-conjugated MCM-41 and KCC-1 for anticancer and antioxidant effects, obtaining higher anticancer effects than with free colchicine [42]. Very recently, we developed a novel DDS for colchicine. The system consisted of KCC-1-functionalized with phosphonate groups and loaded with colchicine, and subsequently coated with folic acid chitosan–glycine complex (MSNsPCOL/CG-FA) [43]. This nanoformulation revealed enhanced selective killing towards cancer cells compared to free colchicine in this order: colon cancer (HCT116) > liver cancer (HepG2) > prostate cancer (PC3). As its cytotoxicity is a major concern, the system is also promising because it exhibits low cytotoxicity (4%) compared to free colchicine (~60%) in normal BJ1 cells. The main mechanism of action was studied in detail for HCT116 cells, indicating primarily intrinsic apoptosis as a result of enhanced antimitotic effects with a contribution of genetic regulation by MALAT 1 and mir-205 and immunotherapy effects by Ang-2 protein and PD-1. 

Loading glabridin, a prodrug compound obtained from the root extract of *Glycyrrhiza glabra,* on MSNs leads to remarkable improvement in its saturation solubility and dissolution velocity [496]. In this context, loading of breviscapine in MSNs significantly improves the solubility and bioavailability [17]. In addition, Ibrahim et al. [183] concluded that incorporating silymarin in MSNs within a lyophilized tablet remarkably increases the dissolution rate and saturation solubility. Similarly, loading of glabridin in MSNs improves the saturation solubility and dissolution velocity [496]. The biological activity, including anticancer activity, of polyphenols and flavonoids obtained from black chokeberry fruits is efficient compared to the free forms when loaded in MCM-41 and ZnO-MCM-41 [497] Co-delivery of topotecan and quercetin by MSNs results in pH-responsive release, subsequently increasing the intracellular release in cancer cells. Ultimately, it induces notable molecular activation (structural changes in tumor cell: endoplasmic reticulum, nucleus, and mitochondria) leading to cancer cell death [264]. Similar evidence has been obtained with targeted delivery of epigallocatechin-3-gallate-loaded MSNs for breast cancer treatment in vivo [266].

## 6. Conclusions and Future Perspective

Engineered MSNs with a variety of nanostructures are important inorganic nanocarriers for drug delivery in nanomedicine applications. MSNs have various unique physiochemical properties, including high pore volume, high specific surface area and porosity. In addition, various organic functional groups can be used for their surface modification by facile processes. MSNs are generally accepted to have good biocompatibility, being safe and showing no-significant side effects. The toxicity of MSNs as in the case of any drug or nanomaterial depends on dose/concentration, material properties, application routes. The degree of toxicity is low as indicated by several studies if the synthesis is performed in optimized conditions or overdose is avoided. Additionally, according to many animal studies, the toxicity of MSNs can diminish by optimizing the synthesis parameters and surface modification. Most in vivo studies generate data stipulating that the suggested average dose of 50 mg/kg is well tolerated in animals and safe without any toxicity or apparent abnormalities. This is considered as an adequate dose to be used, e.g., in cancer therapy. This dose can be increased for oral route administration compared to intraperitoneal or intravenous injection. Importantly, the use of MSN-drug-loaded nanoformulations can allow the use of an even higher dose (three or more times) [174]. As with other nanomaterials, for future translation to clinical applications, the safety aspects of MSNs should be considered carefully for each type because many nanostructures are reported. Recently, the first clinical trial in humans was conducted with oral delivery of fenofibrate formulation based on the ordered mesoporous silica [33]. 

MSNs can be used as multifunctional targeted anticancer delivery systems, delivering a variety of drugs, therapeutic proteins, and antibodies. Furthermore, due to their nanoporous structure, MSNs have a high loading capacity for therapeutic agents and are excellent nanocarriers for internal- and external-responsive release of drugs (e.g., pH, GSH, redox, light, magnetic direction, and ultrasound). The available data indicate that the use of MSNs as prodrug nanocarriers can overcome the present barriers in their application: poor water solubility, low bioavailability, and insufficient targeting. Therefore, the available literature suggests a high potential of MSNs as natural prodrug delivery vehicles. The present pre-clinical and clinical tests show that MSNs are promising drug delivery carriers from a biocompatibility/safety perspective, opening the door towards the clinical nanomedicine application for cancer therapy.

For future research directions, we suggest the importance of co-delivery systems in which two or more anticancer natural prodrugs are combined, as well as exploring thousands of natural prodrugs that have not been thoroughly investigated yet. Furthermore, scientists can investigate loading MSNs with crude extract from plant materials. This can also be explored in synergistic therapy with crude extract containing many prodrug components together. Particularly promising prodrug substances are essential oils applied using MSN-based delivery systems. Their traditional use is only limited to cosmetics and some pharmaceutical applications. The essential oil nanoformulations will add value to cancer therapy. 

The core–shell nanoformulations containing a core of MSNs loaded with prodrugs and a shell of organic substances, such as chitosan, Arabic gum, or others, are highly recommended to establish prodrug delivery systems. As an important parameter, the stability and dispersibility of nanoformulations should be taken into consideration because they affect the biological performance and therapeutic actions. Additionally, we think that the large-scale production for each type of MSNs will lead to obtaining safe material by optimizing and stabilizing the material parameters. In our opinion, animal and reported clinical studies open the doors to develop MSNs-based nanoformulations to be translated into clinical evaluations for cancers soon.

## Figures and Tables

**Table 1:** Classification of porous materials by pore size.

Type of Porosity	Size (nm)
Microporous	<2
Mesoporous	2–50
Macroporous	>50

**Table 2:** The physicochemical properties of the most common mesoporous silica nanoparticles (MSNs) synthesized by various approaches.

Type	BET- Specific Surface Area (m^2^ g^−1)^)	Pore Volume (cm^3^ g^−1^)	Pore Size (nm)	Mechanical Stability (Mpa)	Hydrothermal Stability (°C) (time/h)	Thermal Stability (°C)	Particle Size	Pore Structure	Morphology/ Structure
MCM-41	≥1000	0.7–1.2	1.5–10	86	50	707	~100–200 or microns	Ordered hexagonal	Almost spherical
SBA-15	700–1000	0.75–1.15	5–8	260	100	600	microns	Ordered hexagonal	Rods
KCC-1/DFNS/WMS	~450–1250	0.54–2.18	3–40	216	100	950 or over	50–1100	Disordered	Spherical
Stober silica	~10–350	0.017–0.217	1.2–5.9	NA	NA	NA	20–3000	NA	Solid spheres
KIT					NA	NA	Few microns		
Others	~290–1160	0.85–0.95	~2–10	NA	NA	NA	Few nanometers to microns	Varied	porous

Note: The above-mentioned characteristics of these materials can be controlled and can vary (more or less) from these values. Reproduced with permission from [75], Wiley-VCH Verlag GmbH and Co. KGaA, 2017. NA = not available.

**Table 3:** The biocompatibility, biodistribution, and clearance of MSNs with different shapes, sizes, and surface modifications in vitro or in vivo (injection or oral administration).

MSNs	Study	Biocompatibility	Biodistribution in Organs	References
MSNs (150 to 4000 nm)	Subcutaneous injection Intravenous injections	Good biocompatibility on histological level Death or euthanasia	NA	[139]
Bare, functionalized, polyethylene glycol or hyaluronic acid	In vitro	Biocompatible; do not induce ROS/RNS production; no changes in mitochondrial membrane potential or cell cycle	NA	[141]
MSNs and PEGylated MSNs	Tail vein injection in mice	All treated mice survive well for 1 month after being injected with all MSN and PEG–MSN No pathological abnormality on gross and microscopic histological examinations	Mainly located in liver and spleen; minority in lung, kidney, and heart	[142]
MSNs and solid silica nanoparticles	Lateral tail vein in mice	NA	Liver and spleen due to reticulo-endothelial system; increased accumulation in the lungs due to amine modification; degraded and excreted by urinary and hepatobiliary routes	[143]
MSNs with different shapes	Oral administration in mice	No abnormalities in liver, lung, heart, and spleen; kidneys show particle shape-dependent tissue damage	Liver, lung, spleen, kidney, and intestine Rapidly excreted from feces, and some fraction excreted renally	[144]
MSNs (spheres and rod)	Oral administration in mice	Long-rod MSNs have longer blood circulation than short-rod and spheres	Mainly found in liver and kidney; renal excretion-spherical MSNs cleared faster than rod MSNs	[145]
Multifunctional MSNs	Tail vein injection in mice	Good biocompatibility with low toxicity	Mainly found in liver and spleen Excreted in urine and feces	[21]
Magnetic-doped MSNs	In vitro and in vivo (mouse)	Good biocompatibility	NA	[146]
Biomimetic MSNs	In vitro and in vivo	Biocompatible and no obvious toxicity	Tendency to be biodistributed in brain	[147]
MSNs with different sizes	Intravenous injection in mice	Incidence and severity of inflammatory response was obtained with large size; no abnormal changes obtained for small size	Spleen and liver; clearance in urine and bile depending on size	[148]
MSNs	In vitro and in vivo	No toxicity	Liver and spleen; clearance from urine	[82]

**Table 4:** The toxicity and biosafety of MSNs of various size, shape, surface modification, and route of administration in in vivo studies.

MSNs	Dose	Route of Administration	Period	Toxicity	References
MSNs of 110 nm	Repeated dose at 20, 40, and 80 mg/kg	Intravenous injection in mice	14 days	No death; LD50 of single dose = 1000 mg/kg	[164]
MSNs of 150 nm, 800 nm, and 4 μm	Different doses at single dose	Different routes in rats	3 months	Toxicity depends on route of administration. An amount of 40 mg/kg is safe	[139]
MSNs	40 mg/kg in CD-1 mice	Intravenous injection in mice/rats	14 days	Safe for I.V. administration	[171]
MSNs with aspect ratios of 1, 1.75, and 5	40 mg/kg	Oral administration	14 days	Safe and no changes observed	[144]
MSNs: different sizes and surface modified	Single dose at 25 mg/kg	Lateral tail vein injection	7 days	No clinical toxicity based on histological evaluations; blood biocompatibility	[82]
Functionalized Fe3O4@MSN-PEG and non-modified PEG	40 mg/kg	Intravenous injections in mice	4 days	Non-MSN-PEG caused toxicity to liver, kidney, and spleen tissues; modified PEG nanoparticles showed no toxicity	[172,173]
MSNs and silymarin loaded-MSNs	250 mg/kg	Oral administration in rats	22 days	No evident toxicity in rats	[174]
MSNs	Single dose at 10, 25, and 50 mg/kg	Intraperitoneal application in mice	7 days	No death; almost all tested parameters in liver within normal range	[175]
MSNs of 110 nm	50 mg/kg	Intravenous, hypodermic, intramuscular injection and oral administration in mice	7 days	Caused inflammatory response around the injection sites after intramuscular and hypodermic injection;some toxicity to liver depending on route of application	[29]
MSNs and colloidal silica nanoparticles	2, 20, and 50 mg/kg/day	Intraperitoneal injection in mice	4 weeks	No overt sign of clinical toxicity; some damage to systemic immunity of spleen	[176]
MSNs with different sizes with no surface modification	Single dose	Intravenous administration in female and male BALB/c mice	1 year	No significant changes in body weight, blood cell count, or plasma biomarker indices; no significant changes in post necropsy examination of internal organs and organ-to-body weight ratio;significant liver inflammation and aggregates of histiocytes with neutrophils within the spleen; no chronic toxicity observed	[28]

**Table 5:** Loading capacity for natural prodrugs into MSNs established as recent drug delivery systems for natural medicinal substances.

MSN Type	Surface Modification	Natural Cargo	Loading Content (%)	References
MSNs	Aptamer-functionalized	Curcumin	3.4	[194]
MSNs	Amine-functionalized and chitosan-coated	Gallic acid	Up to 58	[195]
KIT-6	Guanidine-functionalized and PEGylated	Curcumin	50	[196]
KIT-6 and KIL-2	Amino-modified	Curcumin	5–28	[197]
MSNs	Non-modified	Essential oils (lemongrass and clove)	29–36	[198]
MSNs	siRNA, folic acid functionalized	Myricetin	36	[199]
MSNs	Amino-modified	Ursolic acid	22	[200]
MSNs	Non-modified	Curcumin and chrysin	11–14	[201]
MSNs	Non-modified and copolymer-grafted MSNs	Quercetin	3–9.5	[202]
MSNs	Non-modified, amino-functionalized, folic acid-functionalized	Umbelliferone	12–19	[203]
KCC-1 and MCM-41	Folic acid-functionalized	Quercetin, curcumin, colchicine	2–29	[42]
KCC-1	Phosphonate-functionalized	Colchicine	3.5	[43]
MSNs	Non-modified	Thymoquinone	~7.5	[44]
MSNs	Non-modified	Harmine	~45	[204]

**Table 6:** Different loading strategies and their relationships to stimuli release under various conditions for MSNs.

Strategy	Nano System Design	Release	References
Molecular or supramolecular	MSNs-rotaxane	Diffusion under pH	[205,206]
	Pseudorotaxane	Diffusion under redox, pH	[207,208,209]
	Cleavable molecular bridges	Diffusion under plasmonic heating, two-photon irradiation	[210,211]
	Molecular nanovalves	Diffusion under plasmonic heating, various stimuli	[210,212]
Nanoparticles as gatekeepers	Iron oxide nanoparticles	Diffusion under redox, pH	[188,213]
	Cadmium sulfide nanoparticles	Diffusion under redox	[190]
	Gold nanoparticles	Diffusion under pH	[214]
	Zinc nanoparticles	Diffusion under pH	[215]
	Calcium carbonate nanoparticles	Diffusion under pH	[216]
Coatings	Polymer coating	Diffusion under pH	[44]
	Proteins coating	Diffusion under pH	[193]
	Lipid coating	Diffusion under different conditions	[217]

**Table 7:** Some examples of targeted delivery systems for anticancer natural prodrugs using MSNs.

Prodrug	Ligand Used	Cancer Type	In Vitro/In Vivo	References
Berberine	FA	Liver cancer	In vitro and in vivo	[239]
Colchicine	FA	Colon cancer cells	In vitro	[43]
Curcumin	FA	Liver cancer cells	In vitro	[42]
Curcumin	FA	Breast cancer cells	In vitro and in vivo	[260]
Curcumin	FA	Breast cancer cells	In vitro	[261]
Quercetin and doxorubicin co-delivery	HA	Gastric carcinoma	In vivo	[262]
ZD6474 and epigallocatechin gallate	EGFR, VEGFR2, and Akt	Tamoxifen-resistant breast cancer.	In vitro and in vivo	[263]
Topotecan and quercetin co-delivery	Arginine-glycine-aspartic acid (cRGD) peptide	Triple negative breast cancer and multi-drug resistant breast cancer cells (MCF-7)	In vitro	[264]
Quercetin	FA	Breast cancer cells	In vitro	[265]
Epigallocatechin-3-gallate	Peptide	Breast cancer	In vivo	[266]
Anti-miRNA21 and resveratrol co-delivery	HA	Gastric carcinoma	In vitro and in vivo	[267]
Thymoquinone	Whey protein, Arabic gum, or chitosan–stearic acid	Brain cancers	In vitro	[44]
Quercetin	R5 peptide	Colon cancer	In vitro	[268]

FA = folic acid, HA = hyaluronic acid.

**Table 8:** Delivery designs for curcumin in cancer (in vitro/in vivo studies) based on mesoporous silica nanoparticles (MSNs).

Delivery Design	Trigger Release Effect	Cancer Type	Anticancer Mechanism	References
CUR-loaded MSNs incorporated into poly-ε-caprolactone/gelatin (PCL/GEL) hybrid	Sustained release	Human adipose-derived stem cells (hADSCs)	Down-regulation of p16INK4A; up-regulation of hTERT	[326]
SBA-15 doped with silver nanoparticles, coated melanin-like polydopamine, and loaded CUR	pH-sensitive release	Human cervical cancer cells (HeLa) and taxol-resistant non-small cell lung cells (A549/TAX)	NA	[323]
Co-delivery: spiropyran- and fluorinated silane-modified MSNs, loaded doxorubicin and CUR	pH-responsive release	In vivo: HepG2-xenografted mice	NA	[327]
Hollow MSNs, loaded CUR	pH-triggered release	NA	NA	[328]
Co-delivery: PEGylated lipid bilayer coated MSNs, loaded paclitaxel and CUR	Sustained release	In vivo: breast	NA	[324]
Targeted delivery with folic acid-modified MSNs	pH-triggered release	NA	NA	[325]
CUR loaded MSNs	NA	Hepatocellular carcinoma cells (HepG2, liver)		[329]
MSNs functionalized with PEI and loaded CUR	NA	Breast cancers: MCF-7 and MCF-7R cells	Apoptosis: activation of caspase-9, -6, -12, PARP, CHOP, and PTEN; downregulation of survival protein Akt1; downregulation of ER resident protein: IRE1α, PERK, and GRP78	[330]
Targeted delivery: folic acid-conjugated amine-modified MSNs (KCC-1 and MCM-41), loaded CUR	NA	Human hepatocellular carcinoma cells (HepG2) and HeLa cancer cells	Apoptosis: by specific signaling molecular pathways (caspase-3, H2O2, c-MET, and MCL-1)	[42]
Targeted delivery: MSN-modified hyaluronan (HA) or polyethyleneimine-folic acid and loaded CUR	Redox-responsive	Breast cancer cell line (MDA-MB-231). In vivo: mouse xenograft model.	NA	[260]
Targeted delivery: gold nanoparticles immobilized on folic acid-conjugated dendritic MSNs, coated reduced graphene oxide nanosheets, loaded CUR	pH-sensitive and photothermal potency	Human cancer cell lines (MCF-7, human breast carcinoma cells and A549, human lung carcinoma cells)	NA	[261]
Aspartic acid functionalized PEGylated MSNs contained graphene oxide nanohybrid loaded CUR	pH-responsive	Breast cancer MCF-7 cells	NA	[322]
Targeted delivery: folic acid-modified MSNs, loaded CUR	pH-sensitive	Breast cancer MCF-7 cell	NA	[331]
Glycyrrhetinic acid-functionalized MSNs, loaded CUR	NA	Liver hepatocellular carcinoma (HepG2) cells	NA	[332]
Polyethylenimine-modified curcumin-loaded MCM-41	pH-sensitive	Breast cancer MCF-7 cells	NA	[333]
MCM-41 capped by chitosan natural polymer	pH-sensitive	Glioblastoma cancer cell line (U87MG)	NA	[222]
Carboxymethyl cellulose-grafted mesoporous silica hybrid nanogels	NA	Human breast cancer cell line (MDA-MB-231)	NA	[334]
Targeted delivery: CUR-loaded and calcium-doped dendritic MSNs modified with folic acid	pH-responsive	Human breast cancer cells (MCF-7); in vivo animal	Apoptosis: increasing intracellular ROS generation; decreasing mitochondrial membrane potential; enhancing cell cycle retardation at G2/M phase	[335]
Amine-functionalized KIT-6, MSU-2, and MCM-41; loaded CUR	NA	Cancer cells	Apoptosis: generation of intracellular ROS; downregulation of poly-ADP ribose polymerase (PARP) enzyme	[336]
MCM-41 modified, different functionalities, and loaded CUR	Sustained release	Human squamous cell carcinoma cell line (SCC25)	Apoptosis	[124]
KCC-1 and MCM-41 amino-modified and loaded CURC	pH-responsive	NA	NA	[69]

NA = Not applicable, CUR = curcumin.
